# Mapping the epidemiological distribution and incidence of major zoonotic diseases in South Tigray, North Wollo and Ab’ala (Afar), Ethiopia

**DOI:** 10.1371/journal.pone.0209974

**Published:** 2018-12-31

**Authors:** Habtamu Taddele Menghistu, Kassahun Tadesse Hailu, Nigus Abebe Shumye, Yisehak Tsegaye Redda

**Affiliations:** 1 College of Veterinary Medicine, Mekelle University, Mekelle, Ethiopia; 2 Institute of Climate and Society, Mekelle University, Mekelle, Ethiopia; 3 College of Veterinary Medicine, Wollo University, Dessie, Ethiopia; Universidade Nova de Lisboa Instituto de Higiene e Medicina Tropical, PORTUGAL

## Abstract

Zoonotic diseases continue to affect the health and livelihood of resource limited communities. In Ethiopia, despite the presence of a national master plan for prevention, control and elimination of some common zoonotic diseases, well-organized epidemiological data regarding incidence and distribution are lacking. A retrospective cross-sectional study based on a patient medical data recorded from 2012–2016 in selected districts of Southern Tigray, North Wollo zone of Amhara region and Ab’Ala district of Afar region was conducted to map the distribution and Incidence proportion of major zoonotic diseases. The incidence proportion of four major zoonotic diseases (helminthiasis, tuberculosis (TB), rabies and schistosomiasis) was mapped using qGIS software based on the Health Management Information System (HMIS) data collected from district health facilities. The result indicated that, out of a total 1,273,145 observed human disease cases, 53,614 (4.2%) of them were potential zoonotic diseases that include: helminthiasis (51,192), TB (2,085), rabies (227), schistosomiasis (105) and visceral leishmaniasis (7). The highest incidence proportion of TB (262.8 cases per 100,000 population) and rabies (33.2 cases per 100,000 population) were recorded in Gubalafto and Weldya followed by Raya Alamata (253.4 cases per 100,000 population %), and Ab’Ala and Raya Azebo (29 cases each per 100,000 population) for TB and rabies, respectively. The highest incidence proportion for schistosomiasis was reported in Raya Alamata (50.1 cases per 100,000 population) followed by Gubalafto and Weldya (10.8 cases per 100,000 population). The incidence proportion of visceral leishmaniasis per 100,000 population was 4.1, 1.3 and 1.2 cases for Ab’Ala, Gubalafto and Weldiya, and Raya Azebo districts, respectively. Except rabies, which showed high incidence proportion (p<0.0001) in 5–14 age groups, the other zoonotic diseases showed higher incidence proportion (p<0.0001) in age groups above 15 years. Rabies, helminthiasis and schistosomiasis showed statistically significant variation (p<0.0001) among seasons. Rabies and TB showed decreasing trend within the data recorded years. In animals, only 31 rabies cases and 15 anthrax cases were recorded from 2012 to 2016. This finding highlighted the distribution and incidence of some major zoonotic diseases in the study areas. Systematic and detailed research should be conducted in the future to map the distribution of major zoonotic diseases at regional and country level so as to initiate integrated effort from human and animal health authorities and professionals.

## Introduction

In the absence of proper care, the link among humans, animal populations and the surrounding environment can lead to a serious risk of public health with huge economic consequences [1]. This situation is very much pronounced in rural communities of Ethiopia where animals and humans share the same microenvironment including a shelter. A comprehensive review by Cleaveland and colleagues [2] identified 1,400 species of infectious pathogenic organisms to human beings and it includes some viruses and prions (217), bacteria and rickettsia (538), fungi (307), protozoa (66) and helminths (287). Out of these, 868 (61%) were classified as zoonotic diseases originating from animals and 175 pathogenic species were associated with emerging diseases. Among those emerging pathogens, 132 (75%) were zoonotic diseases [2, 3]. Multi-drug resistant tuberculosis (MDR-TB) and rabies are among the emerging zoonotic pathogens with significant economic and public health impact in developing countries [4–7].

In developing countries, every year 2.4 billion zoonotic infections are known to cause widespread illness and 2.2 million human deaths as a result of top 13 major zoonotic diseases [8]. Zoonoses account for over half of all communicable diseases causing illness in humans [8, 9]. These diseases are often endemic and include brucellosis, tuberculosis, salmonellosis, leptospirosis, rabies and others that are under diagnosed, underreported, and which disproportionately affect those who live nearest to animals especially in rural areas of Ethiopia. Even where zoonotic diseases do not cause death, they invariably deepen poverty and destroy livelihoods [10, 11]. Many developing countries like Ethiopia face significant health issues especially zoonotic diseases whose control has been limited by a lack of integrated control measures [12]. In developing countries where resources are limited for the control and prevention of zoonotic diseases, selection and prioritization is important to use these resources efficiently [13, 14]. In this context, use of Geographic Information System (GIS) is of paramount importance to map and study the epidemiological distribution of zoonotic diseases in space and time [15–18].

Even though the epidemiology of major zoonotic diseases in Ethiopia have long been established, to our knowledge there are no available maps of endemic zoonoses to show their spatial distribution and incidence proportion [8, 19]. Mapping the epidemiological distribution and incidence of selected zoonotic diseases will help to identify vulnerable communities where zoonoses pose significant health threats and allocate the limited resources for their control and prevention [9]. Moreover, the inter-sectoral collaboration between different actors (health professionals, veterinarians, environmentalists, and others) is vital for the control and prevention of zoonotic diseases [20]. It is believed that there could be variation of diseases between sex, age, and geographical locations. Thus, the objective of this study was to map the epidemiological distribution and incidence proportion of major zoonotic diseases in selected districts of Southern Tigray, North Wollo of Amhara region and Ab’ala district of Afar region. In the above context, the present study will contribute new data to provide an informed decision-making process leading to better control and prevention of zoonotic diseases in the study areas.

## Materials and methods

### Study areas and populations

**Fig 1:**
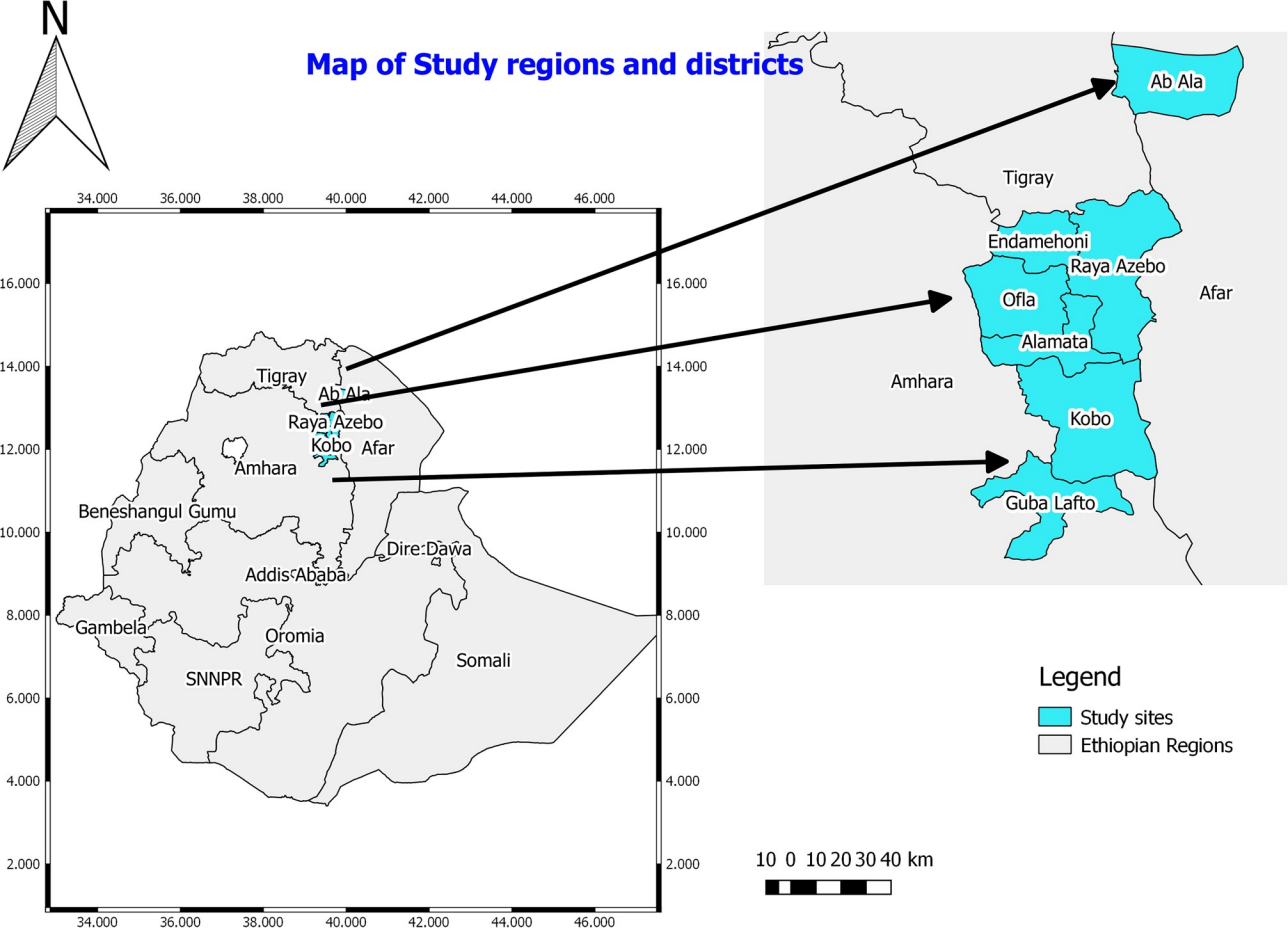
Map of the study regions and districts.

The present study was conducted in three bordering regional states of Ethiopia namely Tigray, Afar and Amhara National Regional states from December 2016 to May 2017. Within these regional states Southern zone, zone two and North Wollo zone were selected purposively from Tigray, Afar and Amhara regions, respectively. Alamata, Raya Azebo, Ofla, and Endamehoni districts were selected from Southern zone of Tigray and Ab’Ala district was selected from zone 2 of Afar region. Whereas, Weldiya, Gubalafto, and Raya kobo districts were included in the present study from South Wollo zone of Amhara region (Fig 1). These eight districts were selected because they are the bordering districts of the three regions where there is high mobility/exchange of people from one district to the other.

The seasonal classification in Ethiopia is mainly based of crop production and rainfall distribution. There are four seasons: i) Summer (‘Kiremt’)–June to August which is characterized by the main rainy season; ii) Autumn (‘Belg’)–September to November which is the harvesting season; iii) Winter (‘Bega’)–December to February which is a dry season; and iv) Spring (‘Tseday’)–March to May characterized by a short rainy season [21].

The human population of the study districts was also collected from the district planning and finance offices in 2016 based on the census conducted in 2007 (Table 1) [22]. The human population data were collected to be used for mapping the epidemiological distribution and incidence proportion of major zoonotic diseases.

**Table 1:** Summary of the human population of the study districts.

District	Total	Male	Female
Ab’Ala	49,205	21,156	28,049
Alamata	149,567	75,018	74,549
Endamehoni	131,560	70,530	61,030
Guba Lafto andWeldiya	231,718	118,152	113,566
Raya Kobo	237,349	119,149	118,200
Ofla	139,622	68,694	70,928
Raya Azebo	161,566	81,572	79,994

Source: District Finance and Planning Offices (CSA, 2007).

### Study design, data collection and analysis strategies

The study was a retrospective cross-sectional study using recorded patient medical data from health facilities.

Recorded data on five zoonotic diseases such as rabies, tuberculosis, schistosomiasis, leishmaniasis and helminthiasis were collected based on the obtained case records (patient medical data) of Health Management Information System (HMIS) data from health centres and health offices, for the years 2012 to 2016 in selected districts. All the health facilities available in each selected district were considered in the data collection. Moreover, data from the regional health bureaus were collected to triangulate the data from the health centres. To avoid potential sources of bias, expert views and literatures were consulted. There is an organized and standardized HMIS data recording system in human health facilities.

Clinical cases presented to the health facilities were diagnosed by physicians and health officers based on their clinical signs and symptoms. For TB and rabies, in addition to the clinical signs and symptoms, suspected specimens were confirmed by laboratory tests. The routine diagnostic approach used for TB at the health facilities was direct smear microscopy and rarely samples were confirmed by regional and national reference laboratories through culture and molecular approaches [23]. Rabid dog bite cases were considered as rabies positive and post-exposure prophylaxis was given to patients. Brain samples from rabid dogs were confirmed by histopathology and direct fluorescent antibody tests at the Ethiopian Public Health Research Institute [24]. Confirmation of schistosomiasis cases was performed by stool examination for the presence of schistosome eggs and in some health facilities, ultrasonography-based approaches were also used to detect for the development of fibrosis which is the typical indication for schistosomiasis cases. Helminthiasis cases were diagnosed in health facilities based on clinical signs and symptoms.

The disease surveillance and control teams in each of the health facilities were responsible for registering disease cases into the HMIS recording format and report them the district health officers. The district health officers also compile the data and report it to the zonal health officers. The zonal health officers report cases to regional health bureau and the regional health bureau report cases to the Federal Ministry of Health. All the disease cases registered are unique and there is no duplication of cases. This is cross-checked by the disease surveillance and control exerts at various levels.

In contrast to this, the data recording system at the veterinary clinics and health posts were found very poor. However, the registered data on the occurrence of animal diseases in the veterinary service centres were used in the present study.

The research team visited each of the health facilities in the study districts and collected the recorded HMIS data after explaining the objectives of the study. The research team also submitted the ethical approval letters from the College of Veterinary Medicine and College of Health Sciences of Mekelle University for conducting the research. The collected data on selected zoonotic diseases were fully anonymized and the researchers had no direct contact with the patients from whom data were collected. TB cases reported in this study were confirmed cases and the patients were under direct observation treatment (DOT) programs. The collected data were entered into a Microsoft Excel sheet and summarized using frequency tables. All the collected patient medical data were analysed anonymously. Besides considering risk factors such as age, sex and seasonal variations, these data were used to map the epidemiological distribution and abundance of major zoonotic diseases in the study districts. The age classification used in the present study was based on the data recording in the health facilities.

A correlation matrix was established using STATA version 15 software to see the association of disease cases in humans and animals. Furthermore, a chi-square test was performed to these the association between disease incidence and risk factors (age, sex and season) and p-value < 0.05 was considered as statistically significant.

### GPS data and application of qGIS for disease mapping

GPS data (altitude, latitude, and longitude) of the study areas were collected from the villages where health centres (health post) and veterinary service centres found together or at least one was available (S1 Table). The application of hand-held GARMIN GPS was used to collect GPS data [25].

The various functionalities of qGIS were used to map the distribution and incidence proportion of four major zoonotic diseases (rabies, tuberculosis, schistosomiasis and helminthiasis) in the study districts using the retrospective data results collected from health facilities (Hospitals, Health centres and Health posts) for the period 2012–2016. To compute a district level distribution of the zoonotic diseases, data-base at district level was created using data obtained from the health centres. The GPS data collected in 1 km radius of the health facilities were used to indicate the location of the facilities in each of the study districts. The ‘Ethio-region’ and ‘Ethio-wereda’ shape files were obtained from the data centre of the Institute of Climate and Society of Mekelle University and used in mapping the epidemiological distribution and abundance of the selected zoonotic diseases. Once the study districts were created from the ‘Ethio-wereda’ shape file, it was saved as a shape file of the study districts. Then organized retrospective data of the zoonotic diseases from the study districts were joined with the study districts shape file applying the ‘joins’ feature of qGIS. Then, disease distribution maps were generated (S1 Protocol).

### Ethical considerations

Ethical clearance for the use of patient medical data was obtained from Mekelle University College of Health Science Health Research Ethics Review Committee (HRERC). Additionally, an ethical approval was obtained from the College of Veterinary Medicine Research and Community Services Council (S1 File). Moreover, the research team had no any direct contact with the patients and used a fully anonymized data recorded by the diseases surveillance and control experts.

## Results

In this study only five zoonotic diseases (rabies, TB, schistosomiasis, helminthiasis, and visceral leishmaniasis (VL)) were found recorded in the health facilities. Of these major zoonotic diseases maps were developed for indicating the distribution and incidence proportion of four of the major zoonotic diseases (rabies, TB, helminthiasis, and schistosomiasis). Since the number of VL cases recorded in the health facilities for the data collection periods was few, the result was summarized in tables. Though the data from Guba Lafto and Weldiya districts were collected independently, they were merged together for mapping purpose.

### Retrospective data from health facilities

Out of the total 1,273,145 recorded disease cases at all health facilities (hospitals, health centres and health posts) in humans from 2012 to 2016, 53,614 (4.2%) cases were composed of five major zoonotic diseases (rabies, TB, helminthiasis, schistosomiasis and leishmaniasis). Out of the total zoonotic diseases, helminthiasis (95.0%) took the greatest share followed by TB (4%). Both forms of leishmaniasis took the least share (Table 2). The year wise distribution of the major zoonotic diseases is summarized in Table 3, where the highest cases for TB (498) and rabies (78) were reported in 2012 in the study areas. The highest cases for schistosomiasis were in 2013 and 2016 where 23 cases were reported in each year. According to the results of the present study, there was an annual decreasing trend of TB and rabies cases during the study period. However, the trend for helminthiasis and schistosomiasis didn’t follow any trend between these years (Table 3).

**Table 2:** Zoonotic disease cases in humans from 2012–2016 in the study districts.

Districts	Rabies		TB		Helminthiasis		Schistosomiasis		VL		Total Zoonoses	
	No.	%	No.	%	No.	%	No.	%	No.	%	No.	%
Ab’Ala	14	6	88	4	3188	6	1	1	2	29	3292	6.14
Raya Alamata	26	11	379	18	10839	21	75	71	0	0	11392	21.24
Endamehoni	18	8	127	6	1426	3	1	1	0	0	1572	2.93
Ofla	25	11	174	8	4773	9	1	1	0	0	4974	9.27
Raya Azebo	47	21	170	8	3827	7	0	0	2	29	4047	7.54
Guba Lafto/Weldya	77	34	609	29	19649	38	25	24	3	43	20287	37.83
Raya Kobo	20	9	538	26	7490	15	2	2	0	0	8050	15.01
Overall	227	0.4	2085	4	51192	95	105		7	0	53614	4.2
Grand Total											1,273,145	100%

**Table 3:** Temporal distribution of zoonotic diseases recorded in human from 2012–2016 in the study districts.

years	Rabies		TB		Helminthiasis		Schistosomiasis		VL		Total case/year
	No.	%	No.	%	No.	%	No.	%	No.	%	
2012	78	34.4	498	23.9%	9875	19.3%	8	7.6%	2	29%	83559
2013	48	21.1%	459	22.0%	10156	19.8%	23	21.9%	1	14%	239818
2014	33	14.5%	411	19.7%	9840	19.2%	28	26.7%	2	28%	277528
2015	32	14.1%	401	19.2%	11276	22.0%	19	18.1%	2	28%	302847
2016	36	15.9%	324	15.5%	7882	15.4%	27	25.7%	2	29%	278590

**NB:** No. Number of cases for each disease; %: Percent share of the disease in the district/year out of the total cases

Seven cases of VL were registered from 2012 to 2016 in humans in all the study districts included in the present study, out of which three cases were recorded in Guba Lafto and Weldiya, and two cases each in Ab’Ala and Raya Azebo (Table 2). Regarding the incidence proportion of this disease per 100,000 population, 4.06, 1.29 and 1.24 cases were registered in Ab’Ala, Gubalafto and Weldiya, and Raya Azebo districts, respectively (S2 Table).

**Fig 2:**
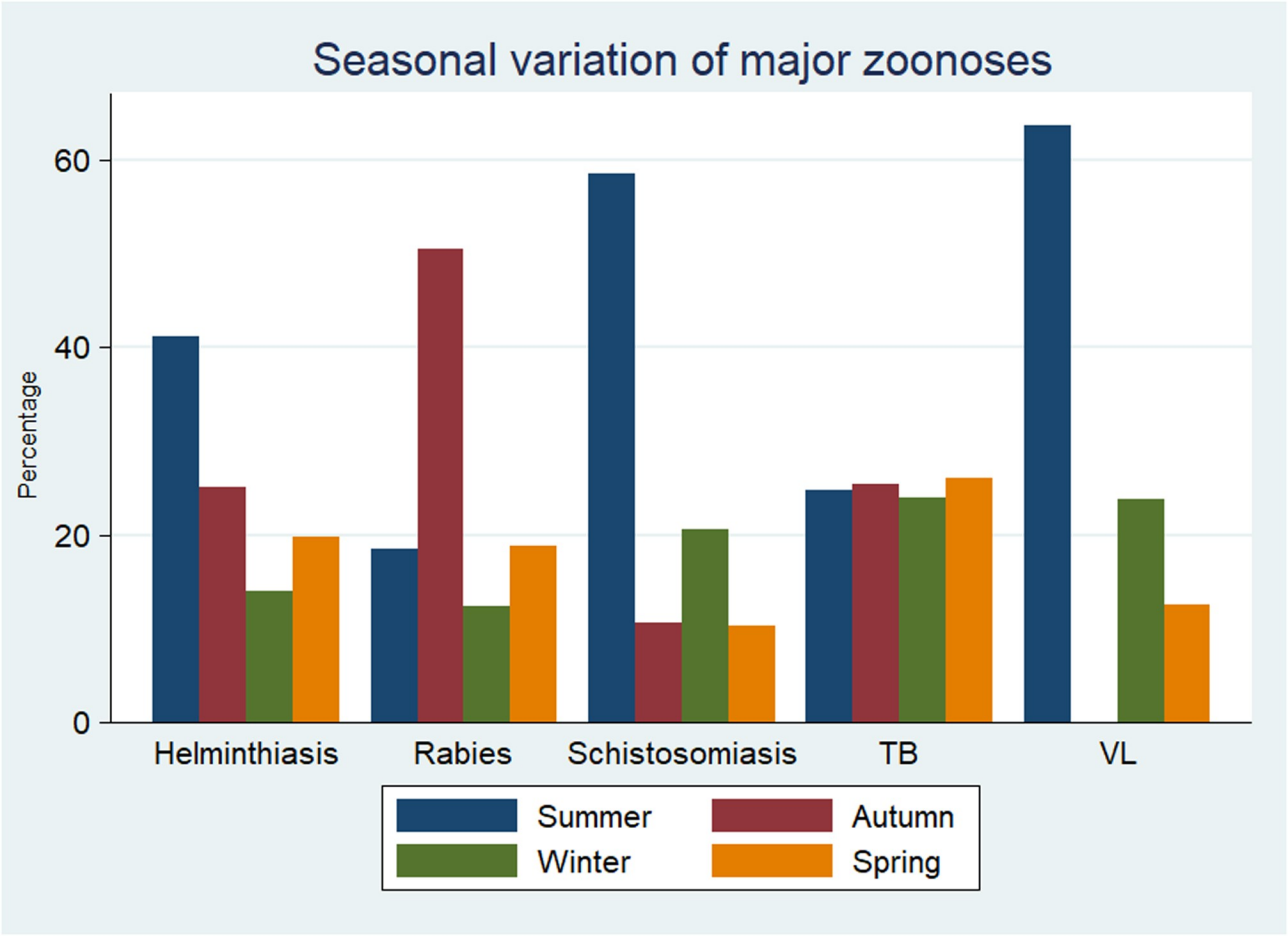
Seasonal occurrence of zoonotic diseases during the five-year period (2012–2016).

Rabies, helminthiasis and schistosomiasis showed a statistically significant (p<0.0001) variation in their seasonal incidence. However, leishmaniasis (p = 0.24) and TB (p = 0.61) didn’t show statistically significant variation in their seasonal occurrence. Rabies cases were higher during the autumn season whereas cases of helminthiasis, schistosomiasis and VL were higher during summer season (Fig 2).

**Fig 3:**
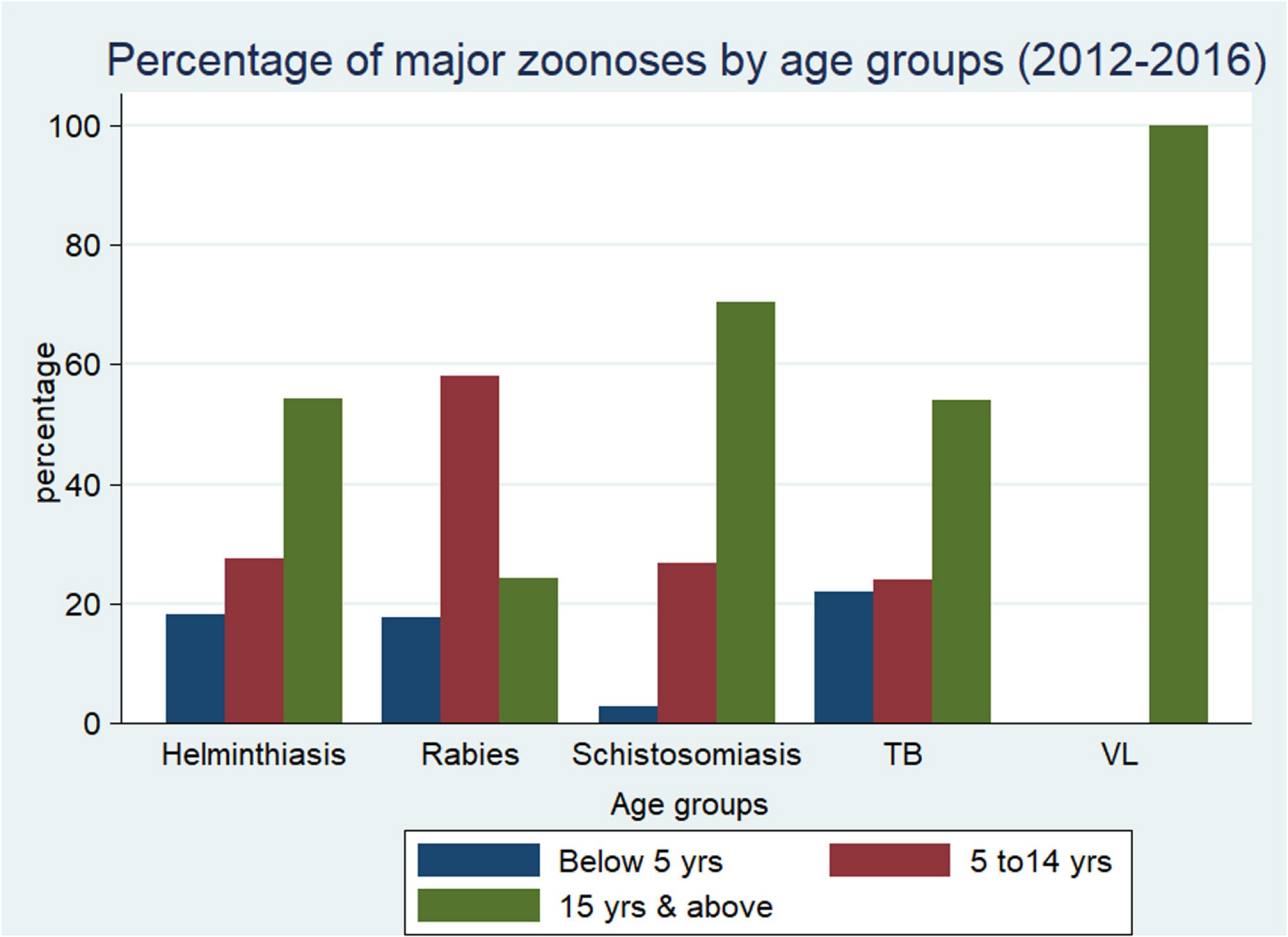
Number of zoonotic disease cases among age groups for the five-year period (2012–2016).

The highest number of zoonotic cases, 5,339.27 cases per 100,000 population (55%) were reported in male individuals as compared to female. Except VL, the other major zoonotic diseases reported in the present study showed a statistically significant difference among males and females. Higher incidence proportion of rabies was reported in females with respect to other diseases (Table 4). About the age group, the highest record of TB (674 cases per 100,000 population), helminthiasis (17,405.5 cases per 100,000 population), schistosomiasis (45.7 cases per 100,000 population), and VL (6.6 cases per 100,000 population) was reported in individuals with the age varying from fifteen and above. However, the highest number of rabies cases were registered in individuals within the age group of 5 to 14 years (Fig 3). All the major zoonotic diseases reported in the study areas showed statistically significant difference (p<0.0001) among the age groups.

**Table 4:** Number of zoonotic disease cases and incidence proportion per 100,000 population by sex for the reporting period (2012–2016).

Sex	Rabies		TB		Helminthiasis		Schistosomiasis		VL		Total zoonoses	
	No.	IP	No.	IP	No.	IP	No.	IP	No	IP	No.	IP
Male	99	17.9	1209	218.1	28207	5089.0	74	13.4	5	0.9	29598	5339.3
Female	128	23.4	936	171.3	22985	4207.3	31	5.7	2	0.4	24083	4408.1
Total	227		2085		51192		105		7		53621	
*p-value*	0.042		<0.0001		<0.0001		<0.0001		0.265			

**NB:** No. Number of cases for each disease; IP: Incidence proportion per 100,000 population.

### Retrospective data from agriculture and animal health offices

The afore-mentioned retrospective data (2012–2016) on six major zoonotic diseases included in the present study were requested from the district agriculture and animal health offices. However, the data obtained were only for rabies and anthrax. Out of the 31 registered rabies cases in different animal species, 11 (35.5%), 9 (29%), 6 (19.4%) and 2 (6.5%) cases were observed in bovine, canine, equines and camel, respectively. Additionally, 3 (9.7%) cases were reported in an outbreak in red foxes in ‘*Key-Amba*’ peasant association of Guba Lafto district (S1 Fig). The maximum number of cases, 9 (29%) of animal rabies were reported from Guba Lafto followed by Raya Azebo, 7 (22.6%) and Ofla, 5 (16%) (Table 5).

**Table 5:** District wise retrospective data of rabies among different species of animals (2012–2016).

District	Total Cases (%)	Number of cases/affected species							
		Canine	popn	Bovine	popn	Equine	popn	Camel	popn
Ab’Ala	2(6.4)	0	738	1(25)	33938	0	7125	1(25)	23069
Raya Alamata	4(12.9)	1(25)	2700	2(50)	123519	1(25)	14793	00	7452
Raya Azebo	7(22.6)	3(42.9)	2159	2(28.6)	195000	1(14.3)	9287	1(14.3)	13589
Ofla	5(16)	1(20)	1672	2(40))	138775	2(40)	17923	00	0.00
Gubalafto	9(29)	3(33.3)	4351	4(44.4)	96316	2(11.1)	19965	00	1289
Raya Kobo	4(12.9)	2(50)	1467	1(25)	213515	1(25)	17473	00	12506
**Total**	**31**	**10 (32.3)**		**12 (38.7)**		**7 (22.5)**		**2 (6.5)**	

NB: Popn.–Population

**Fig 4:**
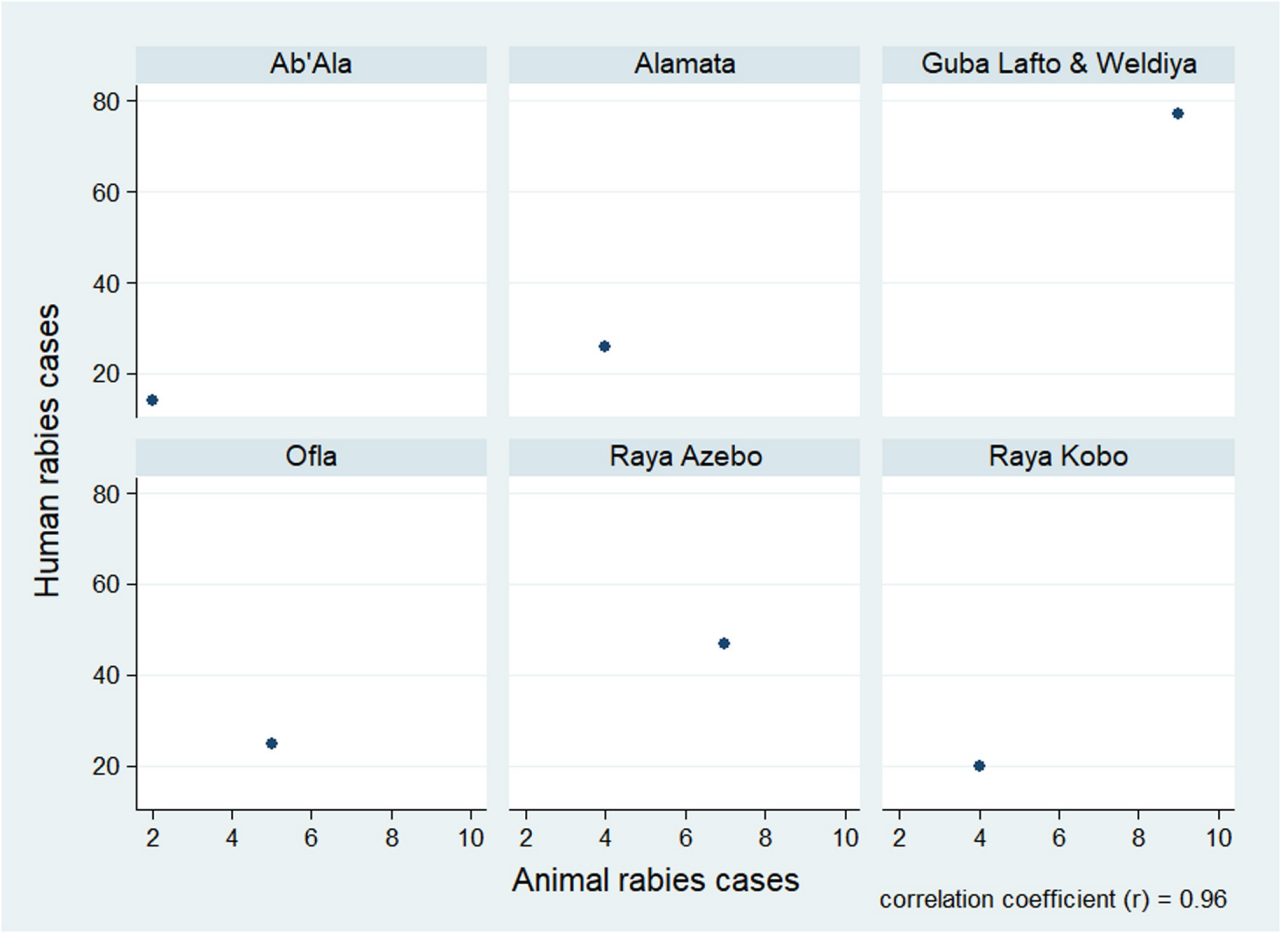
Correlation matrix on rabies cases in humans and animals.

The human cases of rabies were associated strongly and positively (r = 0.96) with rabies cases in animals. In each study district, there was considerable increase in rabies cases in humans as compared to rabies cases in animals (Fig 4).

A total of 15 anthrax cases were reported in different animal species in the study areas from 2012–2016. Out of total recorded cases, 11 cases were observed in bovine and 2 cases each were reported in goats and camel. The highest number of anthrax cases 4 (26.7%) were reported from Guba Lafto, followed by Ray Azebo and Raya Kobo (Table 6). Rabies has the highest number of reported cases as compared to anthrax.

**Table 6:** District wise retrospective data of anthrax among different species of animals (2012–2016).

Districts	Total Cases (%)	No. of affected animals species						Total animal population
		Bovine		Goat		Camel		
		Cases	Popn	Cases	Popn	Cases	Popn	
Ab’Ala	1(6.7)	0	33938	0	149450	1	23069	206457
R/alamata	2(13.3)	2	108519	0	47153	0	7452	163124
R/azebo	3(20)	3	195000	0	42868	0	13589	251457
Ofla	2(13.3)	1	138775	1	64821	0	NI	203596
Gubalafto	4(26.7)	2	96316	1	47154	1	1289	144,759
Rayakobo	3(20)	3	213515	0	113968	0	12506	339,989
Total	15	11	786063	2	315964	2	57905	1,159,932

### Distribution and incidence proportion of selected zoonotic diseases

**Fig 5:**
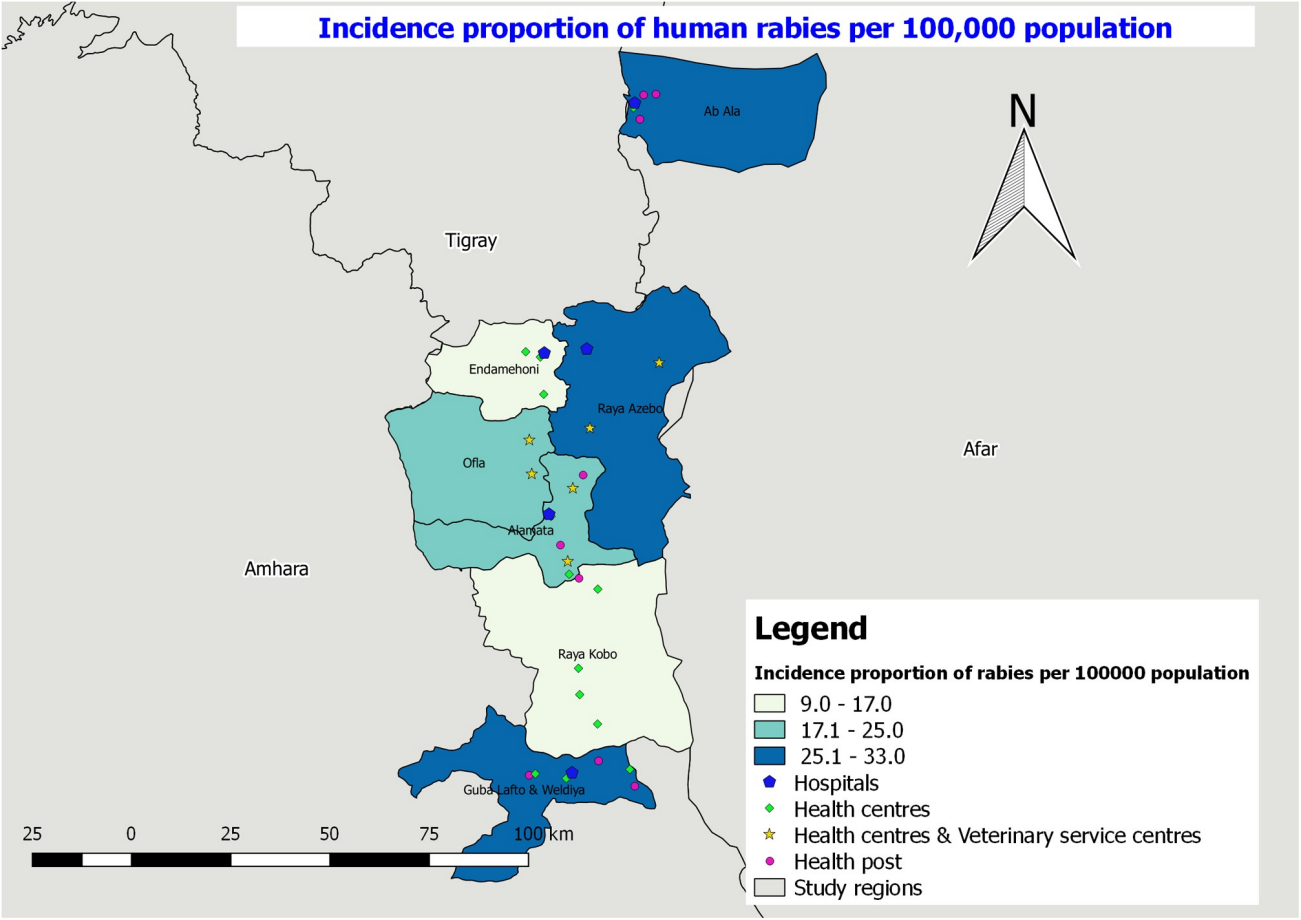
Incidence proportion of human rabies per 100,000 population in the study districts from 2012–2016.

**Fig 6:**
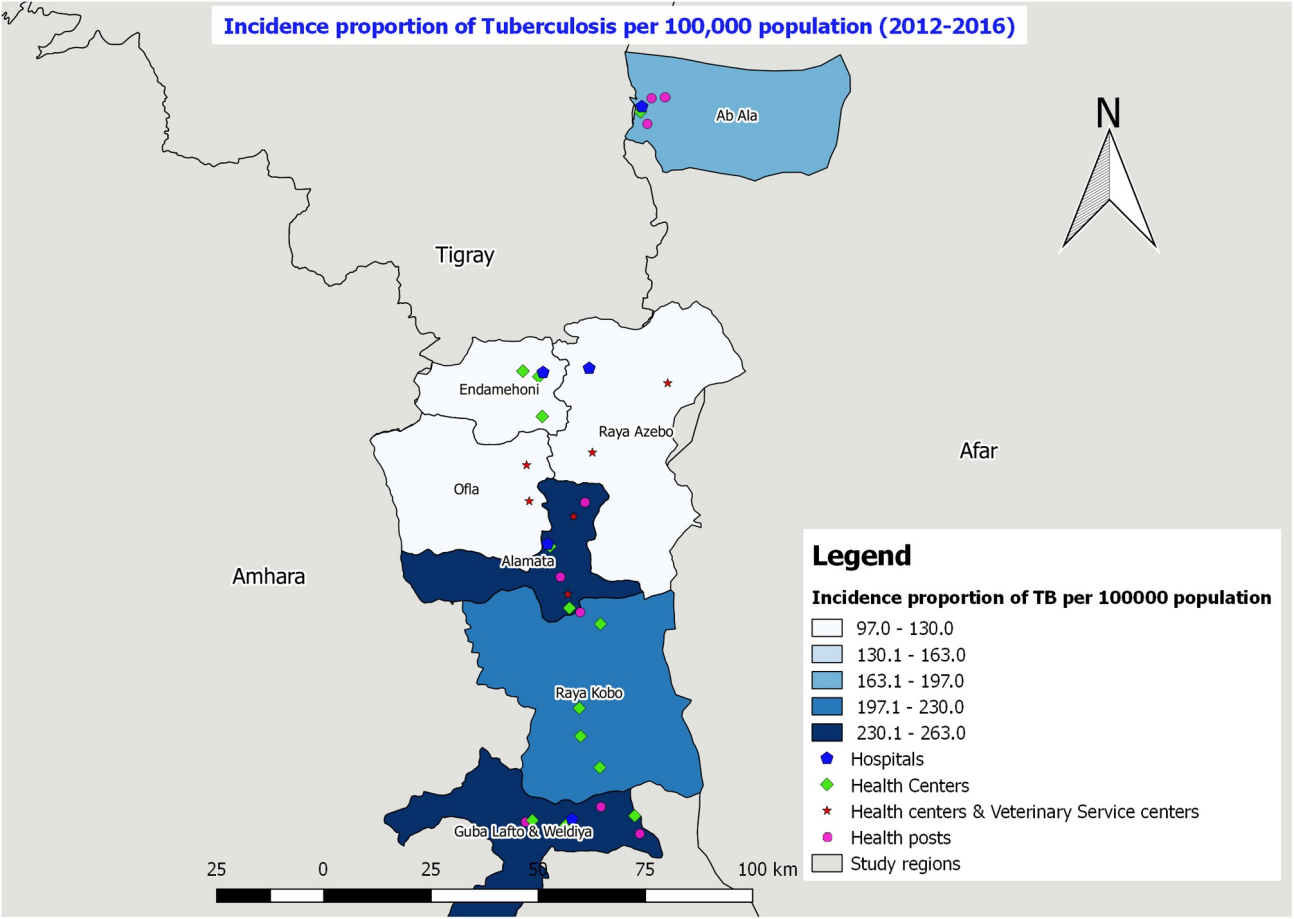
Incidence proportion of tuberculosis per 100,000 population in the study districts from 2012–2016.

**Fig 7:**
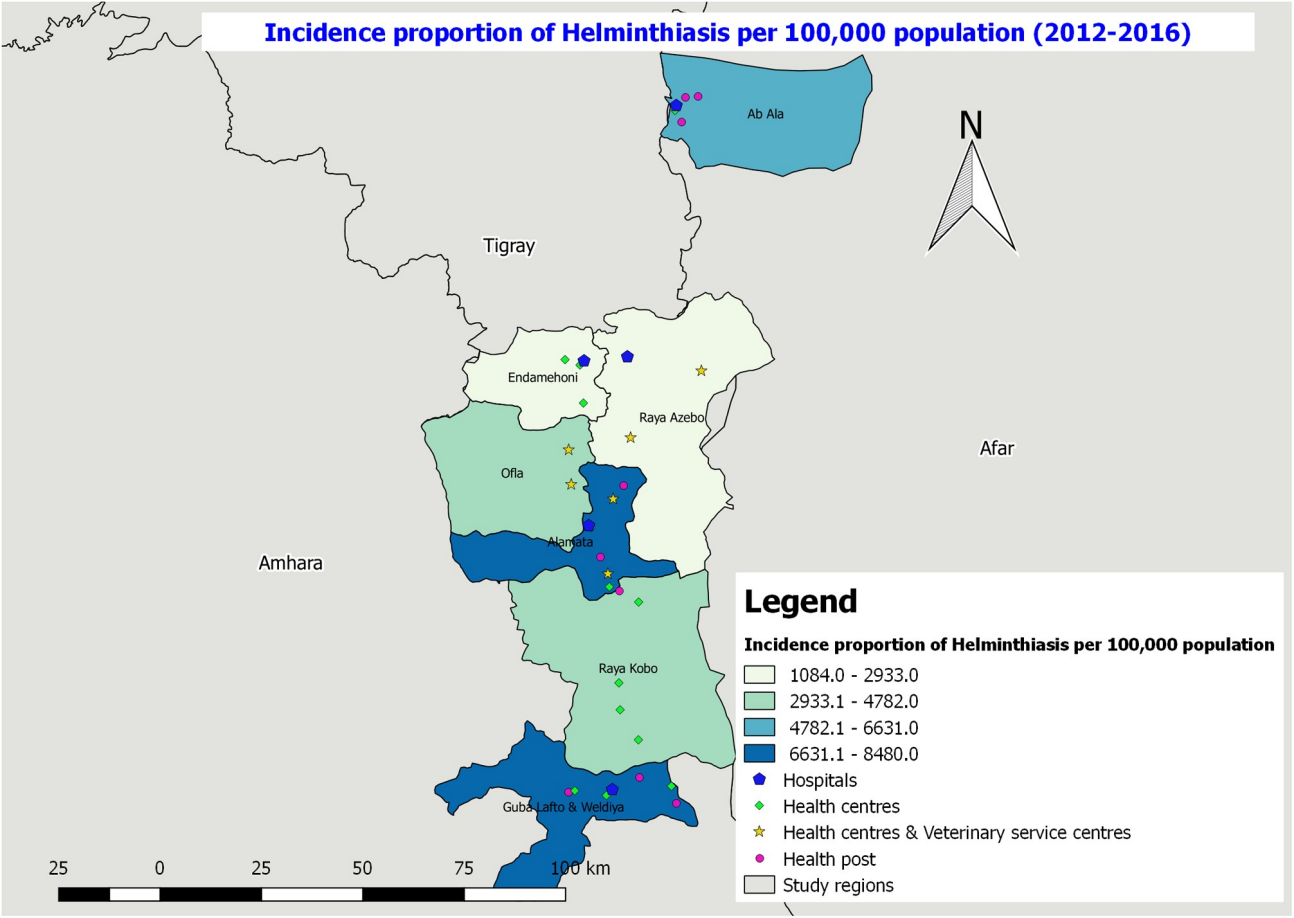
Incidence proportion of helminthiasis per 100,000 population in the study districts from 2012–2016.

**Fig 8:**
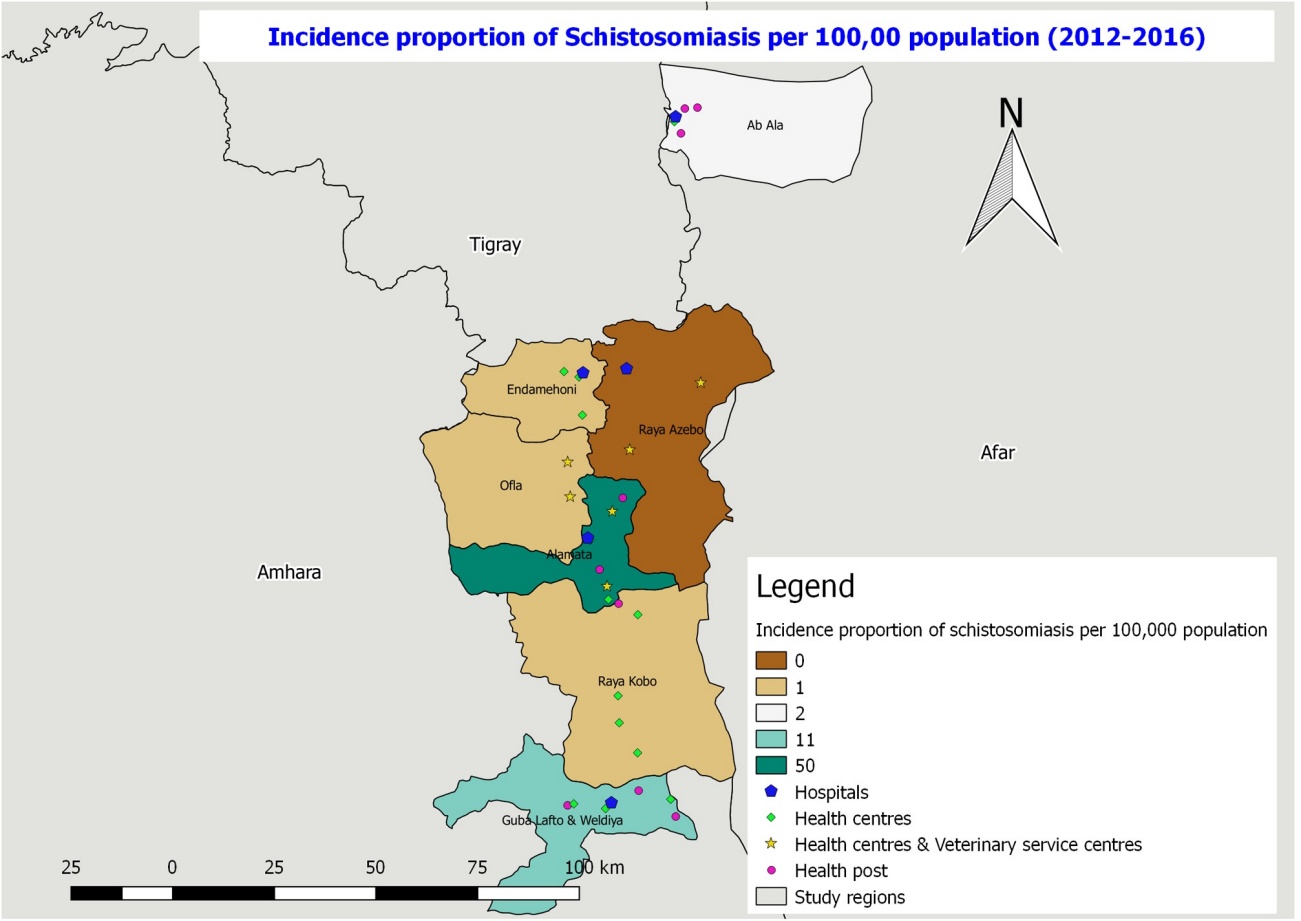
Incidence proportion of schistosomiasis per 100,000 population in the study districts from 2012–2016.

Epidemiological distribution and incidence proportion for the four major zoonotic diseases were mapped using the functionalities of qGIS software and the results are presented in Figs 5–8. The highest incidence of rabies (25.1–33.0 cases per 100,000 population) from 2012–2016 was reported from Guba Lafto and Weldiya, Ab’Ala and Raya Azebo districts and the lowest incidence proportion (9.0–17.0 cases per 100,000 population) were reported from Endamehoni and Raya Kobo districts (Fig 5).

The highest incidence of tuberculosis was recorded in Guba Lafto and Weldiya, and Raya Alamata (230.1–263.0 cases per 100,000 population) followed by Raya Kobo (197.1–230.0 cases per 100,000 population) (Fig 6).

Among the total observed zoonotic diseases the highest proportion (95%) of zoonotic disease was helminthiasis (Table 2). The highest number of helminthiasis cases were registered in the health facilities of Guba Lafto andWeldiya, and Raya Alamata districts (6631.1–8480.0 cases per 100,000 population) followed by Ab’Ala district (4782.1–6631.0 cases per 100,000 population) (Fig 7). With regard to the incidence proportion of schistosomiasis, the highest cases were registered in Raya Alamata district health facilities (50 cases per 100,000 population) followed by Guba Lafto and Weldiya (11 cases per 100,000 population). The other district health facilities reported less number of cases for the reporting period (Fig 8).

**Fig 9:**
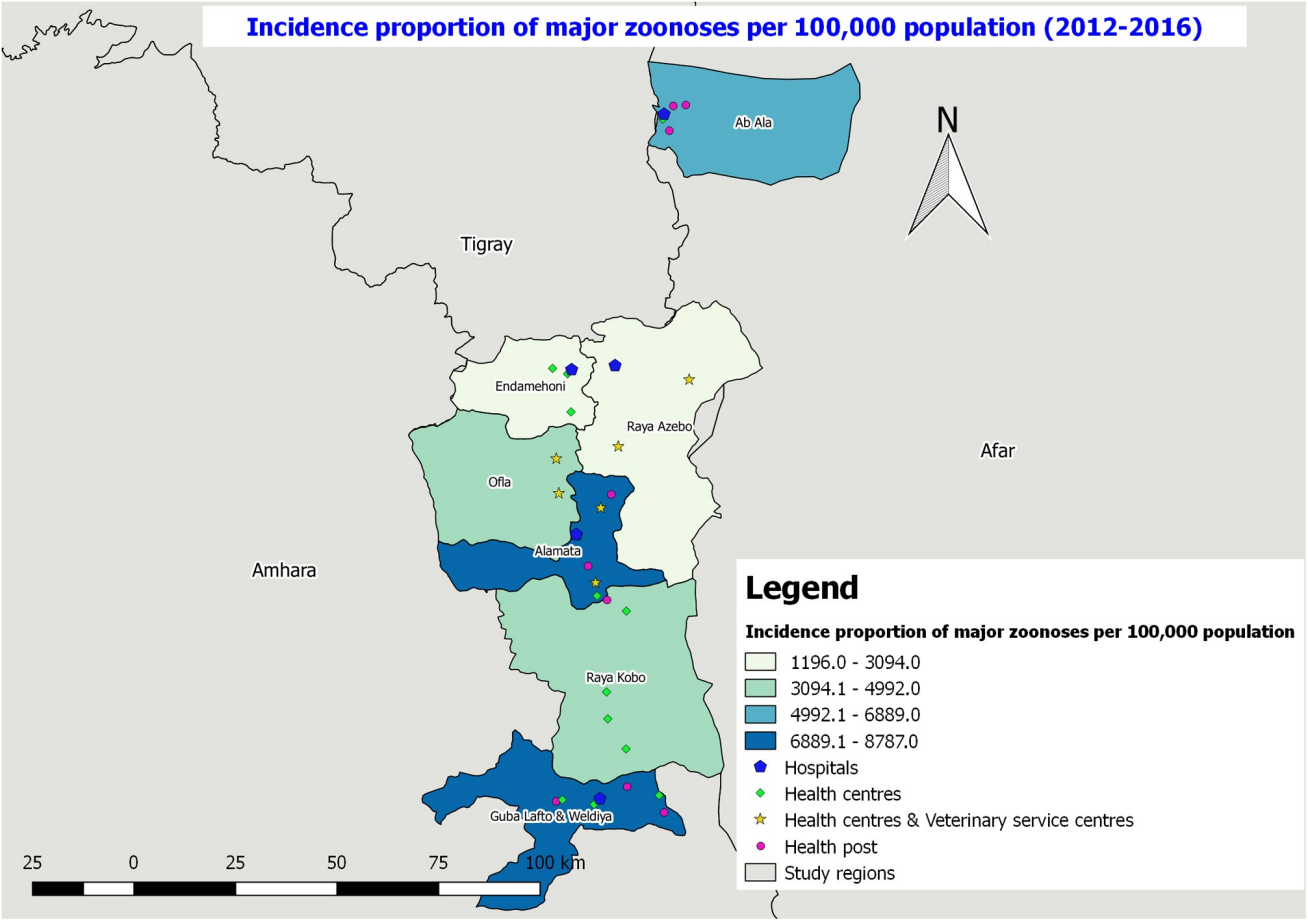
Overall incidence proportion of major zoonotic diseases per 100,000 populations in the study districts from 2012–2016.

With regard to the overall incidence proportion and distribution of four major zoonotic diseases (rabies, tuberculosis, helminthiasis and schistosomiasis) commonly registered in the health facilities of the districts during the reporting period, the highest incidence proportion and distribution was reported in Gubalafto and Weldya, and Raya Alamata districts (6889–8787 cases per 100,000 population) followed by Ab’Ala district (4992–6889 cases per 100,000 population) (Fig 9).

## Discussion

In this study, an attempt was made to map the epidemiological distribution and burden of the afore-mentioned major zoonotic diseases which have a public health importance using qGIS based on the data obtained from health facilities. According to this study, out of the total human disease cases registered in the health facilities for the reporting period, 4.2% of them were five major zoonotic diseases (i.e. helminthiasis, TB, rabies, schistosomiasis, and VL). There was an annual decreasing trend in TB and rabies cases from 2012 to 2016. However, the trend for helminthiasis and schistosomiasis didn’t follow any trend between these years. The decrease in the cases of TB and rabies through the years could be due to the continued health promotion services and measures undertaken by the government in controlling these diseases. The increase in the awareness of the community could also play a role for the decreasing trend in these diseases [26–29].

In the present study, the number of helminthiasis cases from the retrospective data were highest as compared to other diseases. The major helminthiasis cases that have public health significance could be taeniasis, ascariasis, trichuriasis and hydatidosis. These findings are also supported by previous prevalence estimates of different helminthiasis cases, which bears the highest burden among sub-Saharan African countries [30, 31]. A study conducted by Yami and colleagues [32] reported 47.1% intestinal helminthiasis especially *Ascaris lumbricoides* and hookworm in Jimma zone among school children. The probable reason for the high burden of helminthiasis cases in the study districts could be associated with poor socio-economic status and poor environmental sanitation [32]. In addition, the consumption of raw animal meat could contribute to the high incidence of helminthiasis [33]. The highest incidence of helminthiasis cases in males above 15 years old during the summer (rainy) season could be associated with intensive engagement in farming activity, and availability of warmth and moisture environment that enhance the development of free-living infective stages of helminth parasites [34]. Moreover, during this season there are high chances of contamination of water sources due to flood.

The highest incidence proportion of tuberculosis was recorded in Guba Lafto and Weldiya, and Raya Alamata districts. This could also be associated with the existing habits of drinking raw milk in the study areas among other factors. The close contact of animals with humans especially in rural areas and the prevailing low standard of hygiene in the production of farm animals are potential risk factors that favour the spreading of infections from animals to humans. Several researchers also highlighted supporting reasonings for the possible spread of bovine tuberculosis (BTB) from animals to humans [7, 35]. Legesse et al. [35] also described raw milk, which is a major food source for the rural communities and considered as the major source of *Mycobacterium bovis* infection. The possible justification for higher cases of TB in males above 15 years old in the present study could be due higher mobility of males as a breadwinner compared to females, which may increase their exposure to the disease.

There is a higher report of rabies in humans than animals. The reported cases of rabies in animals are very low and this could be associated with weak reporting system in the animal sector. However, there is a strong and positive correlation between human and animal rabies cases, which shows the zoonotic importance of the disease. Compared to the present study a higher number of rabies cases (2798 cases) in humans were reported by in selected districts in Tigray region [36]. A study by Habtamu and colleagues [37] indicated higher number of cases of human rabies (4729) and 44 deaths in Tigray region for the period of 2009–2012. Abebe and his colleagues [38] also reported a total of 154 and 62 suspected animal and human rabies cases, respectively in Northwest Tigray. Out of the total rabies cases, the highest incidence proportion was registered in Gubalafto and Weldya, Ab’Ala and Raya Azebo. This may be due to high dog population, poor trend of the communities to vaccinate their dogs and lack of awareness about this disease. Individuals within the age groups of 5 and 14 were more affected by rabies. This could be associated with the fact that children of this age group have a close contact with dogs and there is lack of awareness about the disease [38]. The higher incidence of rabies during autumn season is associated with suitable breeding season of dogs in Ethiopia which favours the transmission of the disease.

The highest incidence of schistosomiasis were registered in Raya Alamata district followed by GubaLafto and Weldiya district. The possible reason for the high number of cases in Raya Alamata could be due to the availability of water bodies and swampy areas which are suitable for the snail. Abebe et al. [39] also reported high cases of schistosomiasis in Waja-Timuga peasant association of Raya Alamata district where the highest cases were reported in individuals with the age of 15 years and above. Similarly Assis and his co-workers [40] also reported higher cases of schistosomiasis in individuals aged above 15 years old. The higher incidence of schistosomiasis during summer (rainy) season might be associated with the availability of suitable environment for the vector and parasite [39].

A total of 15 anthrax cases were reported in different animal species in the study areas, however, there was no registered case of anthrax in human. This result is very low as compared to previous studies conducted around Tanqua-Abergelle district where 504 and 2680 anthrax cases in animals and human, respectively were reported [41]. However, the absence of anthrax cases doesn’t indicate for the absence of the disease in humans but the culture of reporting by the community is low and human HMIS recording system doesn’t include anthrax in the reporting template.

## Conclusion

Even though disease burden estimations were based on limited, underreported and incomplete data, however, the current mapping indicated that there is a significant incidence and distribution of helminthiasis, tuberculosis, rabies, and schistosomiasis. The HMIS data recording format should be adopted to enhance the reporting practice in the veterinary service sector. However, the HMIS needs also updating to include some major zoonotic diseases like anthrax and specific helminthiasis infections (taeniasis, hydatidosis, ascariasis, trichuriasis, etc). Effective and well-organized control measures should be developed with a special focus on identified risk factors and areas. Moreover, the incidence proportion of the diseases should be taken into account while designing control and prevention measures. Furthermore, there is a need for an integrated zoonotic disease surveillance system in the country applying the one health approach so as to minimize the impact of these diseases in humans and animals.

## Limitations of the study

The present study was conducted in selected districts of three national regional states of Ethiopia. Due to the limited number of districts involved, the findings might not directly reflect the geographical distribution and incidence of major zoonoses at regional level. Moreover, the inconsistency in disease reporting and diagnosis might also influence the overall results. The other limitation of this study was due to the variability among recruited health facilities (hospitals, health centres, health posts and veterinary service centres) in their infrastructure and human capacity, and the variability in diagnostic tests within and between diseases in both humans and animals. All the above factors might affect the number of cases in the study areas (i.e. there could be under-representation of cases).

## Supporting information

S1 File Letter of ethical approval. (PDF)

S1 STROBE Checklist STROBE checklist. (DOC)

S1 Fig A fox died of rabies infection in Guba Lafto district. (JPG)

S1 Protocol Procedures for mapping the distribution and incidence of major zoonotic diseases using qGIS software. (PDF)

S1 Table GPS data for health facilities of the study districts. (XLSX)

S2 Table Actual number of cases and incidence proportion of major zoonotic diseases per 100,000 population. (XLSX)
