# Correction: Mapping the epidemiological distribution and incidence of major zoonotic diseases in South Tigray, North Wollo and Ab'ala (Afar), Ethiopia

**DOI:** 10.1371/journal.pone.0211292

**Published:** 2019-01-17

**Authors:** The PLOS ONE Staff

There are several errors in the headings for Tables 3 and 4. The publisher apologizes for the errors. Please see the corrected Tables 3 and 4 here.

**Table 3:** Temporal distribution of zoonotic diseases recorded in human from 2012–2016 in the study districts.

years	Rabies		TB		Helminthiasis		Schistosomiasis		VL		Total case/year
	No.	%	No.	%	No.	%	No.	%	No.	%	
2012	78	34.4	498	23.9%	9875	19.3%	8	7.6%	2	29%	83559
2013	48	21.1%	459	22.0%	10156	19.8%	23	21.9%	1	14%	239818
2014	33	14.5%	411	19.7%	9840	19.2%	28	26.7%	2	28%	277528
2015	32	14.1%	401	19.2%	11276	22.0%	19	18.1%	2	28%	302847
2016	36	15.9%	324	15.5%	7882	15.4%	27	25.7%	2	29%	278590

**NB:** No. Number of cases for each disease; %: Percent share of the disease in the district/year out of the total cases

**Table 4:** Number of zoonotic disease cases and incidence proportion per 100,000 population by sex for the reporting period (2012–2016).

Sex	Rabies		TB		Helminthiasis		Schistosomiasis		VL		Total zoonoses	
	No.	IP	No.	IP	No.	IP	No.	IP	No	IP	No.	IP
Male	99	17.9	1209	218.1	28207	5089.0	74	13.4	5	0.9	29598	5339.3
Female	128	23.4	936	171.3	22985	4207.3	31	5.7	2	0.4	24083	4408.1
Total	227		2085		51192		105		7		53621	
*p-value*	0.042		<0.0001		<0.0001		<0.0001		0.265			

**NB:** No. Number of cases for each disease; IP: Incidence proportion per 100,000 population

## References

[1] Menghistu HT, Hailu KT, Shumye NA, Redda YT (2018) Mapping the epidemiological distribution and incidence of major zoonotic diseases in South Tigray, North Wollo and Ab’ala (Afar), Ethiopia. PLoS ONE 13(12): e0209974 10.1371/journal.pone.0209974 30596744 PMC6312287

