# Supplementary material for: Mapping the epidemiological distribution and incidence of major zoonotic diseases in South Tigray, North Wollo and Ab’ala (Afar), Ethiopia
Source: PLoS One. 2018 Dec 31;13(12):e0209974. doi: 10.1371/journal.pone.0209974 (PMC6312287; doi:10.1371/journal.pone.0209974)
Supplement: S1 File — (PDF) [file pone.0209974.s001.pdf]

Ethical Clearance from Mekelle University College of Health science

Mekelle University
College of Health Sciences
Health Research Ethics Review Committee (HRERC)

To: Kassahun Tadesse
Principal Investigator
Mekelle

Date: 13/07/2016

RE: **Notification of Expedited Approval**

ERC 0795/2016

Protocol: Mapping the distribution and abundance of major zoonotic diseases using geographic information system and remote sensing in south Tigray, Ethiopia

Dear PI

This is your notification that your above referenced study has received **EXPEDITED APPROVAL** on **13/07/2016**. This ethics review approval will expire on **12/07/2017**.

The research study cited above has been reviewed and it has been determined that it meets the criteria for expedited review. The HRERC will be apprised of this decision at its monthly meeting.

The PI should comply with national and international scientific and ethical guidelines. Any reportable events (serious adverse events, breaches of confidentiality, protocol deviation or protocol violations) or issues resulting from this study should be reported immediately to the HRERC. Any amendments (changes to any portion of this research protocol including but not limited to protocol or informed consent changes) must have HRERC approval before being implemented.

All correspondences and inquires concerning this research protocol must include the ERC number, the name of the PI and the protocol title.

Sincerely,

CC:

- Chief executive Director
  Mekelle University

Commencing health research without approval is unethical!

Ethical Clearance from Mekelle University College of Veterinary Medicine

Mekelle University
College of Veterinary Medicine
Research and Community
Service Office

መቐለ ዩኒቨርሲቲ
የእንስሳት ሕክምና ኮሌጅ
የምርምርና ማህበረሰብ አገልግሎት
ጽ/ቤት

Ref. No: CVM-CRC/54/08
Date: July 7/2016

To: Dr. Kassahun Tadesse
Principal Investigator, MSc student
Department of Veterinary Medicine,
Mekelle University-College of Veterinary Medicine

RE: **Notification of Exempt Approval**

It is to be recalled that you requested the Research and Community Service Office (RCSO) of College of Veterinary Medicine to get exemption approval for the research project entitled ***"Mapping the Distribution and Abundance of Major Zoonotic Diseases using Geographical Information System and Remote Sensing in Southern Zone of Tigray, Ethiopia"***, selected to be fund by OHCEA.

Therefore, this is your notification that your above mentioned topic has received **EXPEDITED APPROVAL** on **June 21, 2016**. This ethics review approval will expire on **November 30, 2016**. The research study cited above has been reviewed and it has been determined that it does not have serious ethical concern on animal subjects.

It is stated that every procedure that the researchers need to follow during the interaction with animals should be in line with research ethics principles and procedures of the College and OIE. Amendments (changes) must have RCSO's approval before being implemented. All correspondences and inquires concerning this research protocol must include the RCS council reference letter, project topic, the names of the PI and team members.

Sincerely,

[illegible]
Yohannes Tek[illegible]
RCSC Chairman

CC:
- Dean, College of Veterinary Medicine
  Department Head, Veterinary Medicine
  MU, Kalamino Campus

☎ + 251-344401619, Mobile: +251-912-015594 ✉ 231 Mekelle, Ethiopia Fax + 251-04-409304
Email: john.asfaw@yahoo.com

ጤነኛ እንስሳ ለጤነኛ ኢኮኖሚና ማ/ሰብ!!! Health Animal for Health Economy and Community!!!
