# Supplementary material for: Mapping the epidemiological distribution and incidence of major zoonotic diseases in South Tigray, North Wollo and Ab’ala (Afar), Ethiopia
Source: PLoS One. 2018 Dec 31;13(12):e0209974. doi: 10.1371/journal.pone.0209974 (PMC6312287; doi:10.1371/journal.pone.0209974)
Supplement: S1 STROBE Checklist — (DOC) [file pone.0209974.s002.doc]

S1 Checklist: STROBE Checklist

|  | Item No | Recommendation |
| --- | --- | --- |
| **Title and abstract** | 1 | Title, short tile |
|  |  | Abstract, paragraph 1 |
| Introduction | | |
| Background/rationale | 2 | Introduction, Paragraph 1-3 |
| Objectives | 3 | Introduction, Paragraph 3 |
| Methods | | |
| Study design | 4 | Methods, Paragraph 5 |
| Setting | 5 | Methods, Paragraph 1-4 |
| Participants | 6 | Methods, Paragraph 6 |
| Variables | 7 | Methods, Paragraph 6 |
| Data sources/ measurement | 8* | Methods, Paragraph 6 |
| Bias | 9 | Methods, Paragraph 6 |
| Study size | 10 |  |
| Quantitative variables | 11 | Methods, Paragraph 6 |
| Statistical methods | 12 | Methods, Paragraph 6 - 8 |
| Results | | |
| Participants | 13* |  |
| Descriptive data | 14* | Results, Paragraph 2 |
|  |  |  |
| Outcome data | 15* | Results, Paragraph 2-8 |
| Main results | 16 | Results, Paragraph 2-10 |
|  |  |  |
|  |  |  |
| Other analyses | 17 |  |
| Discussion | | |
| Key results | 18 | Discussion, Paragraph 1 |
| Limitations | 19 | Conclusion, Paragraph 1 |
| Interpretation | 20 | Discussion, Paragraph 2-6 |
| Generalisability | 21 | Conclusion, Paragraph 1 |
| Other information | | |
| Funding | 22 | Acknowledgements, Paragraph 1 |
