# Supplementary figures and images for: Mapping the epidemiological distribution and incidence of major zoonotic diseases in South Tigray, North Wollo and Ab’ala (Afar), Ethiopia
Source: PLoS One. 2018 Dec 31;13(12):e0209974. doi: 10.1371/journal.pone.0209974 (PMC6312287; doi:10.1371/journal.pone.0209974)

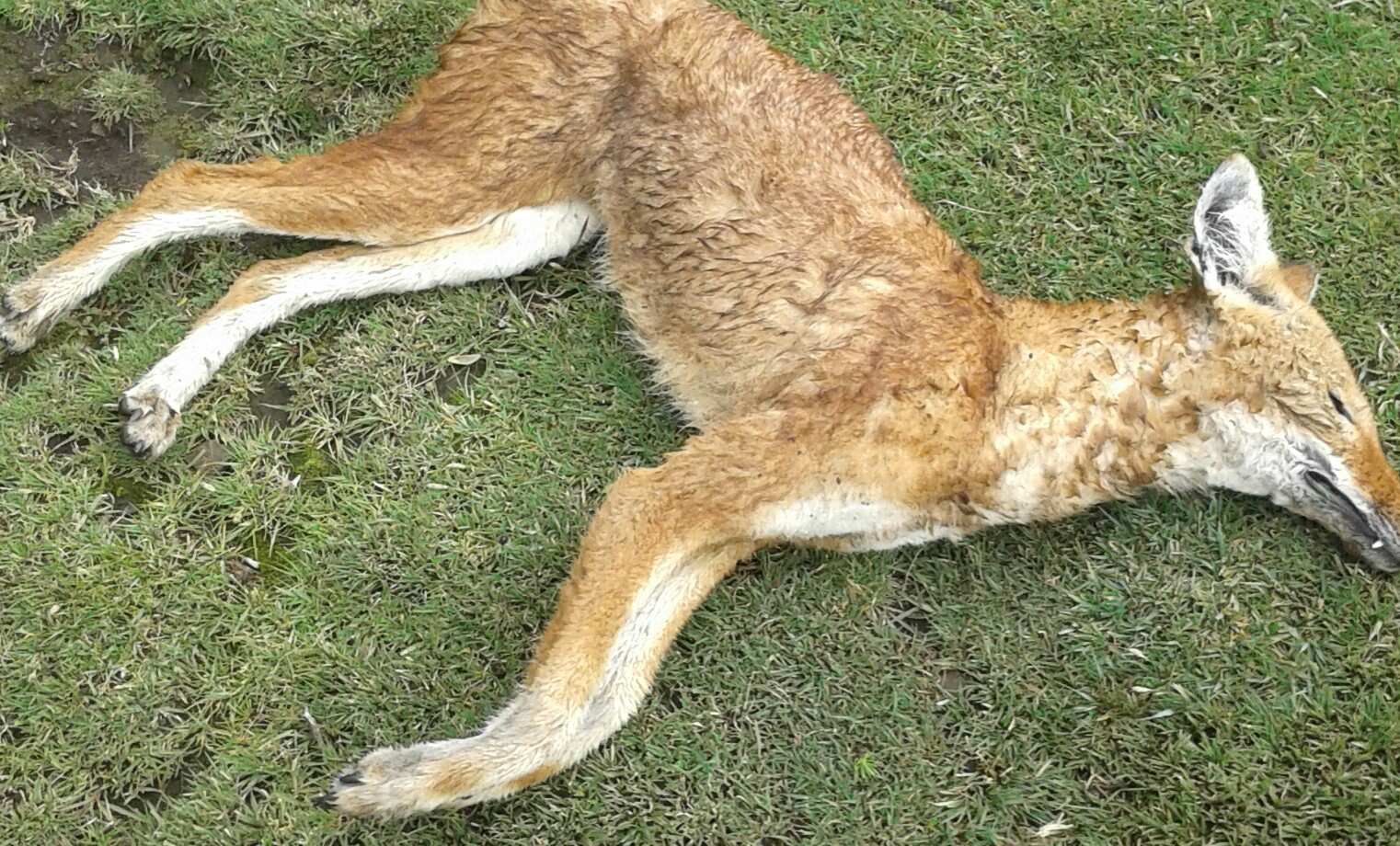

Supplement: S1 Fig — (JPG) [file pone.0209974.s003.jpg]
