# Supplementary material for: Mapping the epidemiological distribution and incidence of major zoonotic diseases in South Tigray, North Wollo and Ab’ala (Afar), Ethiopia
Source: PLoS One. 2018 Dec 31;13(12):e0209974. doi: 10.1371/journal.pone.0209974 (PMC6312287; doi:10.1371/journal.pone.0209974)
Supplement: S1 Protocol — (PDF) [file pone.0209974.s004.pdf]

Procedures for mapping the distribution and incidence of major zoonotic diseases using qGIS software

1. To launch QGIS, click: Start -> All Programs -> QGIS -> QGIS. QGIS normally adds the version number after “QGIS”, in our case we used version 2.18.3. The main windows of QGIS can be divided into three regions Controls and menus, table of contents, and Data view window) as shown below.

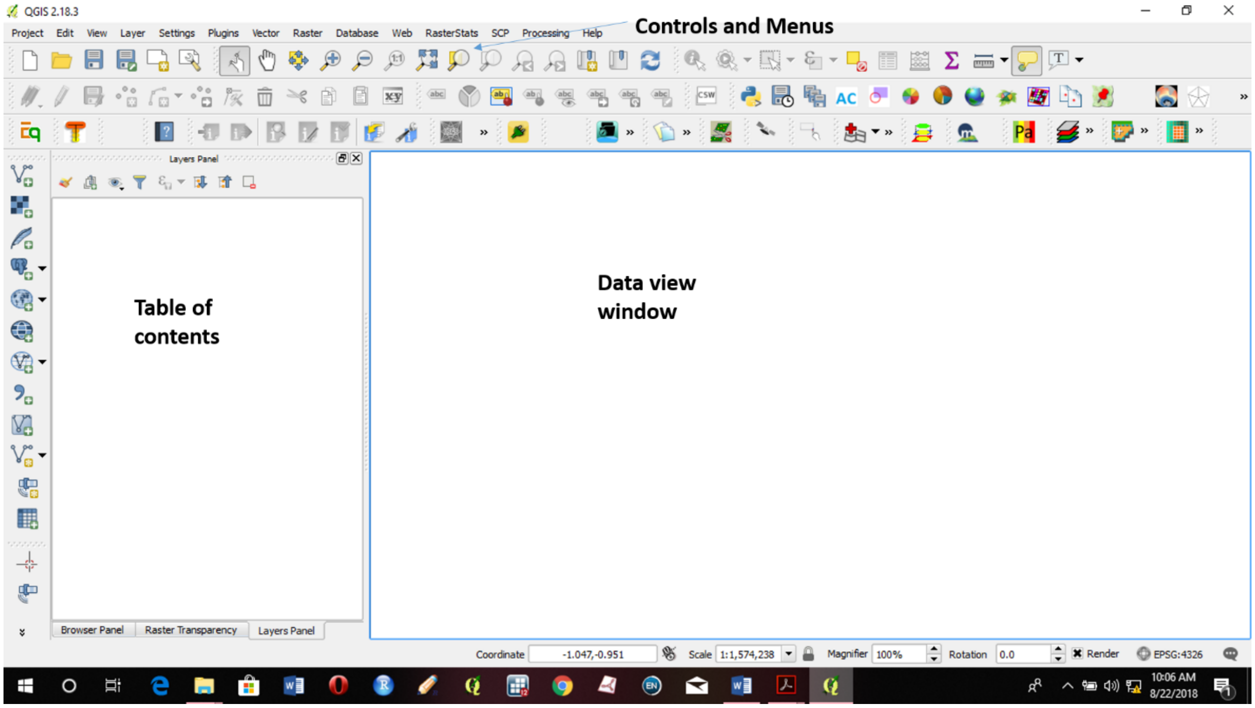

2. To load a vector dataset of your interest, click on the **Layer>Add Layer and, Add Vector Layer** 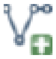 or simply press **ctrl+shift+v.** This will bring up a new dialog:

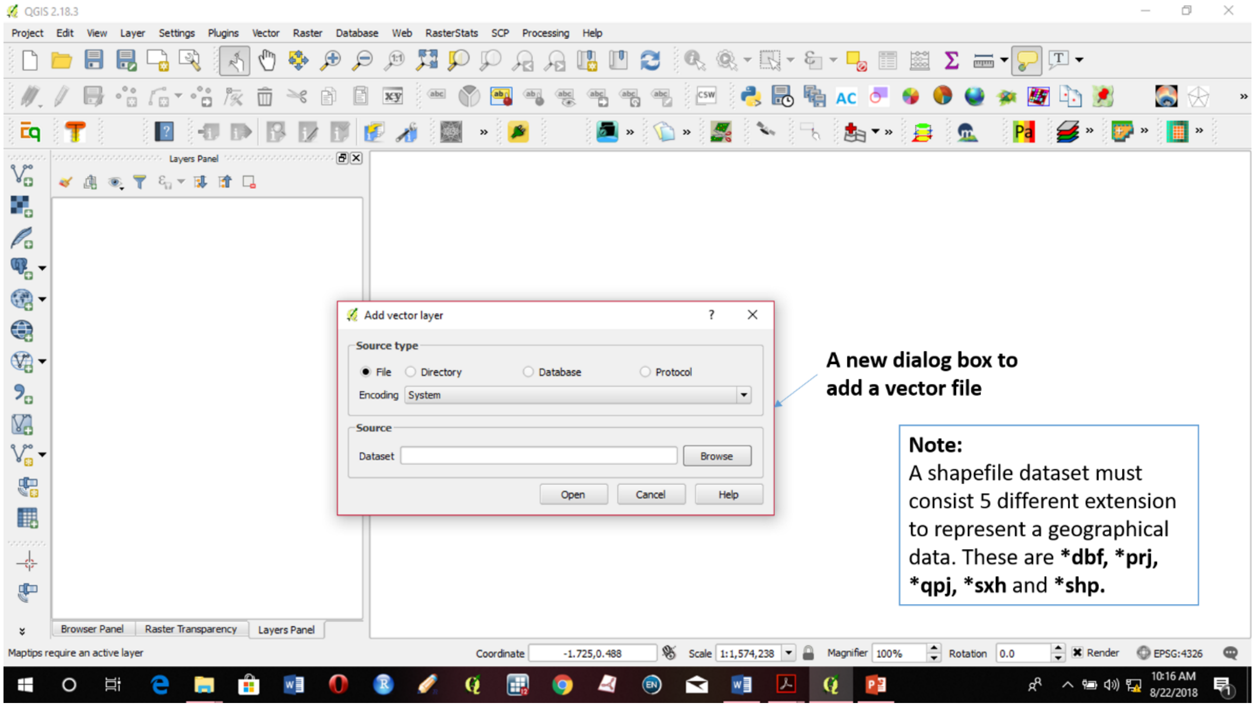

When File option is selected as Source type, hit Browse, and look for the file of your interest (in our case "Ethio_Region.shp") from the place you saved in

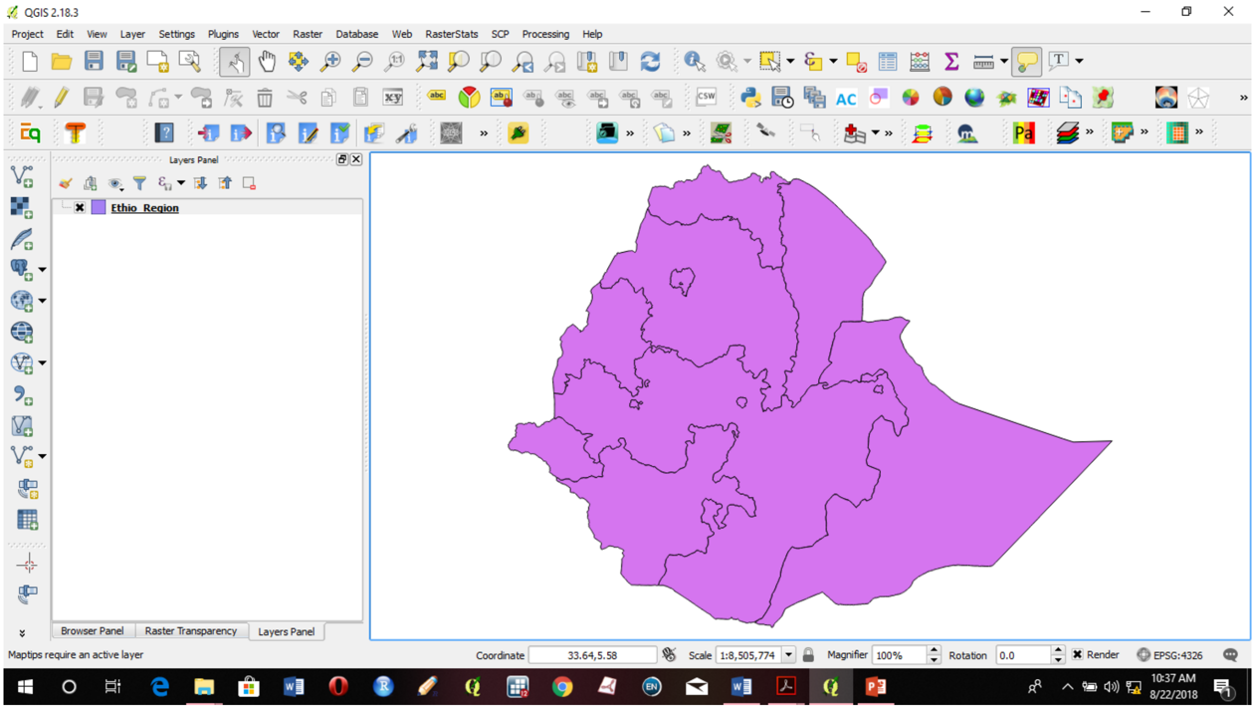

3. Repeat step 2 if you have other shape files to be incorporated to the map. *In our case we did this for study districts shape files ("Study sites.shp") and health facility shape files in our study districts ("Health centers "HC.shp", Health centers and veterinary service centers "HC_VSC.shp" "Health post(HP).shp" and "Hospitals;shp")*
   To have a good view of the study districts and health facility shape files, **zoom to full extent menu** as indicated below. To label the districts and the regions, **right click** on shape file from the Map Content area > scroll down and click on **properties > Labels > Show label for this layer > Label with > "WOREDANAME" > Apply > OK**.

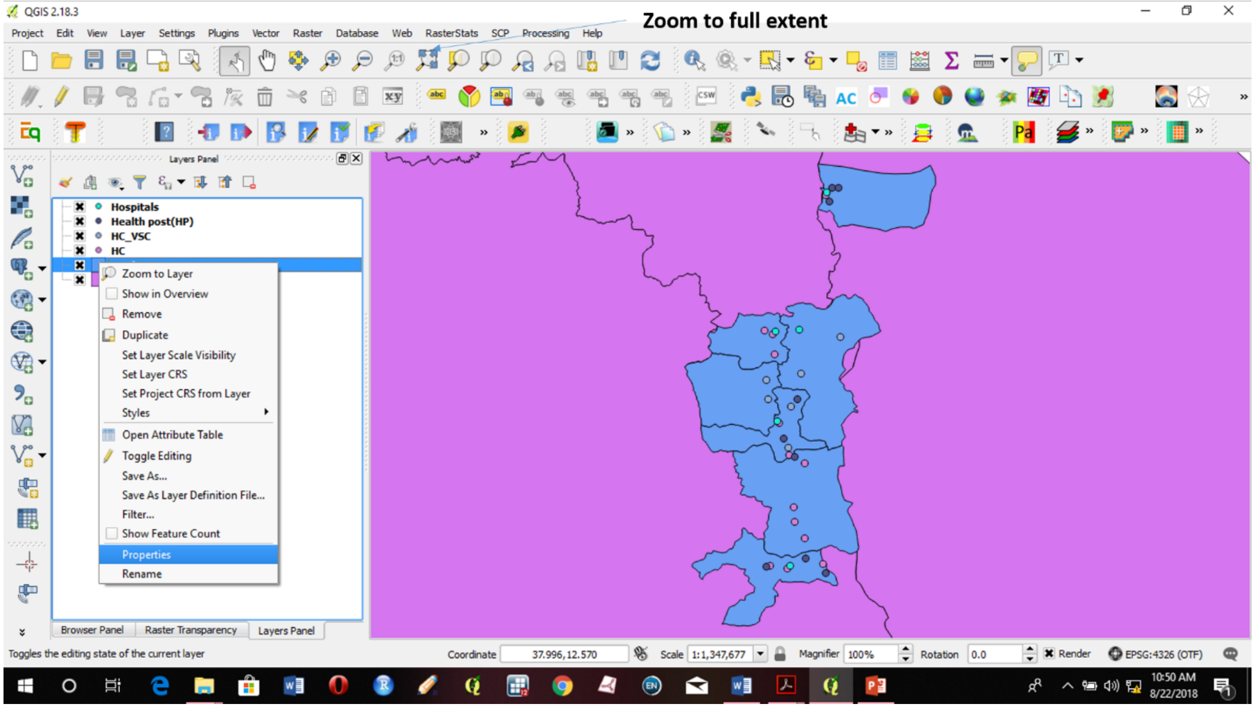

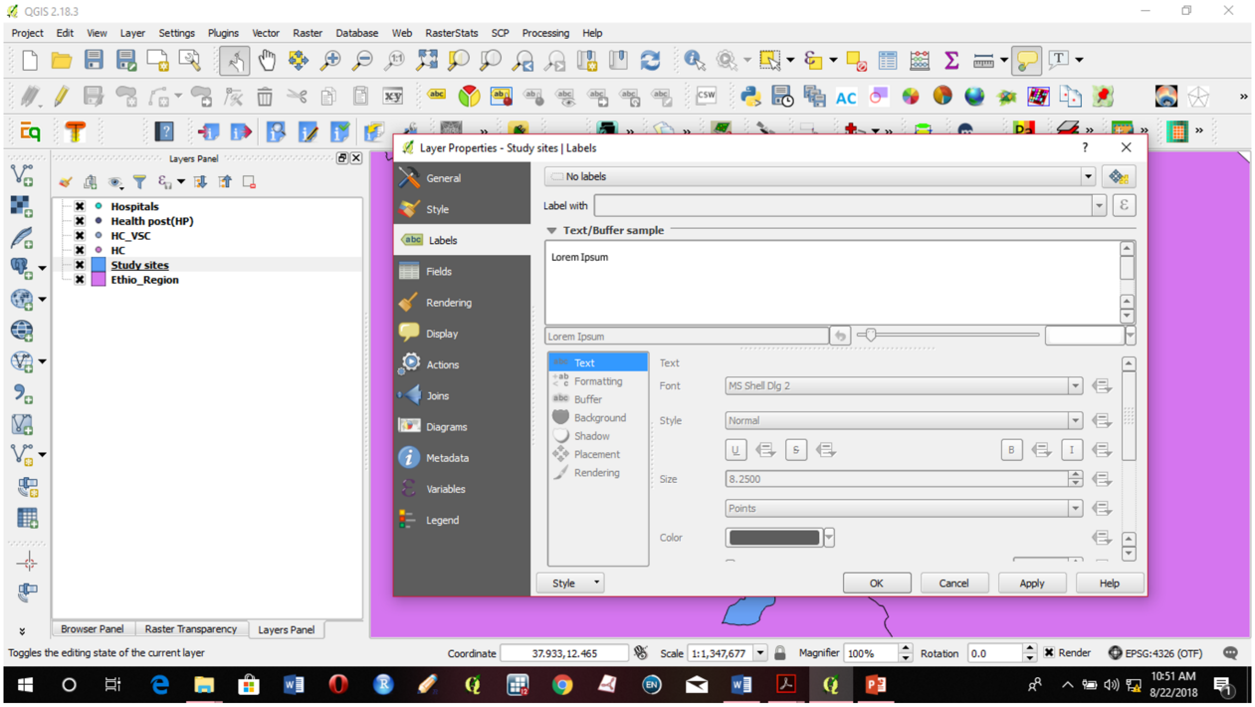

Similarly repeat this for the regions. You can also change the shape of the GPS points of the health facilities: **Right click** on the shape file from the Map Content area **"HC" > Properties > Style > Scroll down to "symbols in group" > Select the symbol > Apply > OK**. It is also possible to change the background color for the shape files: **Right click** on the shape file from the Map Content area **"Ethio_regions" > Properties > Style > Color > Select the color > Apply > OK**.

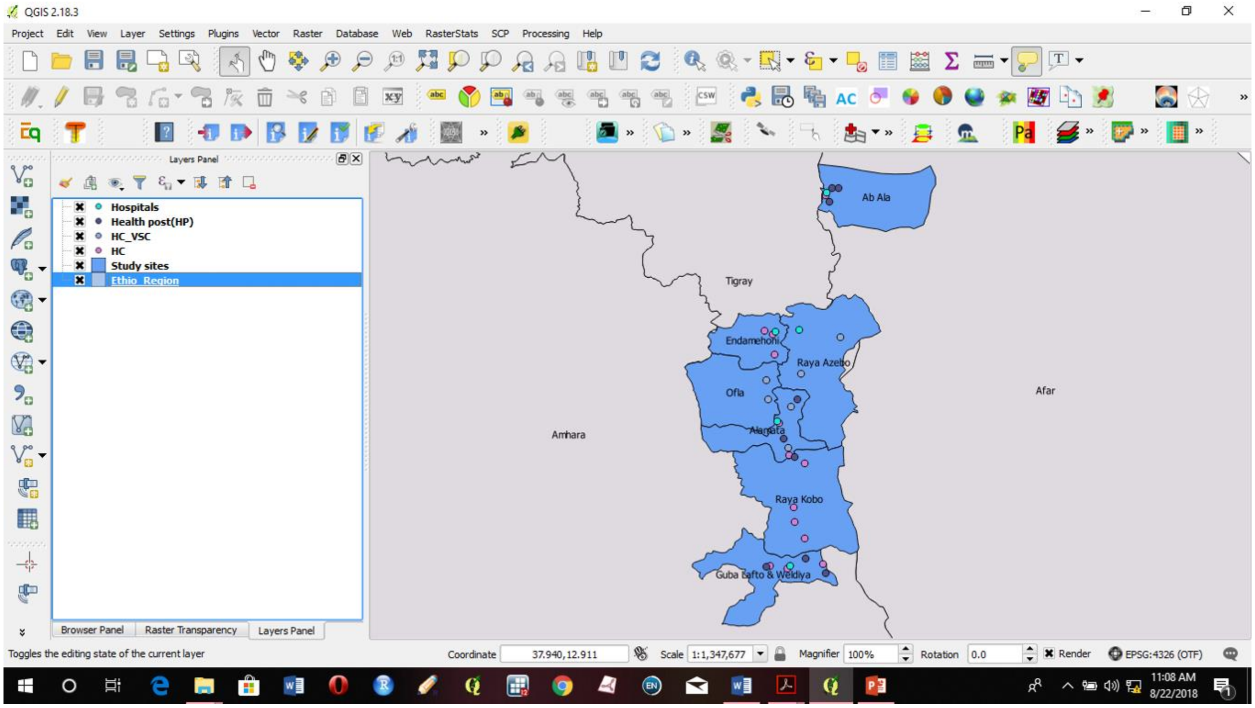

4. Create a table that consisted the incidence rate of zoonotic diseases in an excel sheet and save it as comma delimited 'csv' file format. To add a spreadsheet into QGIS, **click on the Layer>Add Layer and click Add Delimited Text Layer**. This will bring up a new dialog:

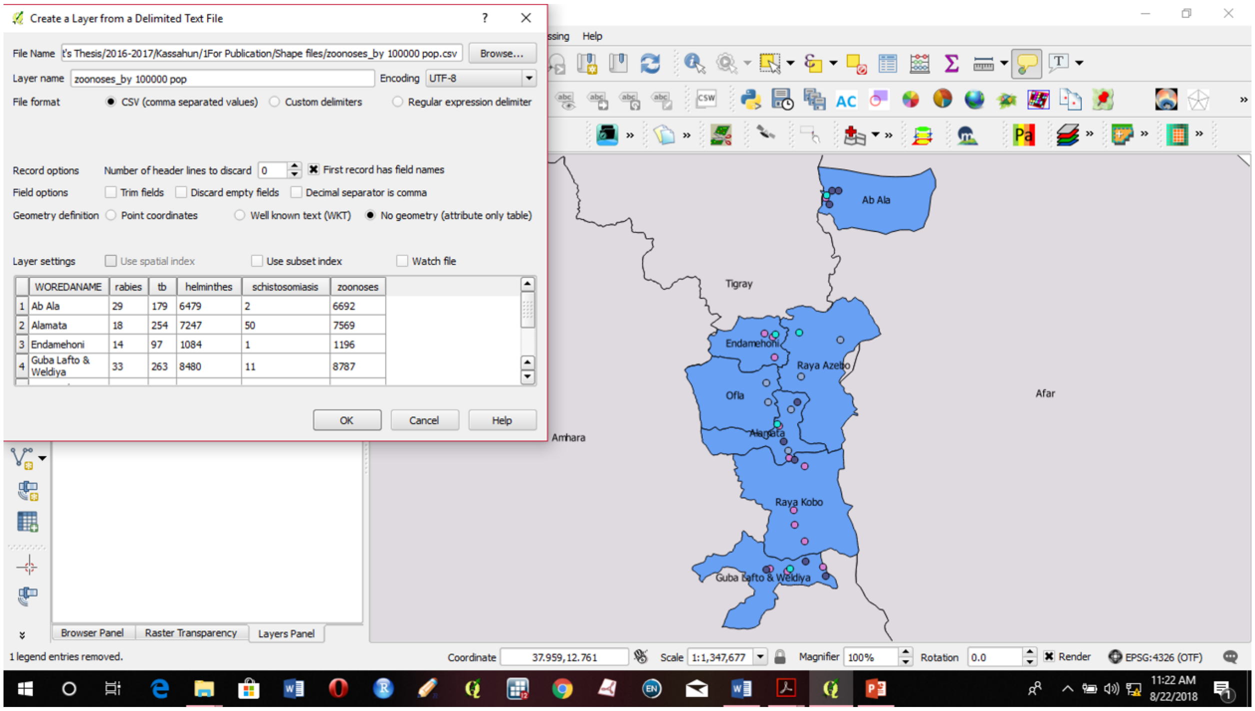

This dialog allows you to select a wide range of properties for your layer. Even though most common usage of the delimited file is comma, there are always different options. If your file using another delimiter rather than a comma, you can define your custom delimiter from Custom delimiters.

Hit OK to add your data into QGIS.

5. Right click the data layer from the **Map Content area**, then click **Open Attribute Table** to view attributes of the data.

zoonoses_by 100000 pop :: Features total: 7, filtered: 7, selected: 0

	WOREDANAME	rabies	tb	helminthes	schistosomiasis	zoonoses
1	Ab Ala	29	179	6479	2	6692
2	Alamata	18	254	7247	50	7569
3	Endamehoni	14	97	1084	1	1196
4	Guba Lafto & We...	33	263	8480	11	8787
5	Raya Kobo	9	227	3156	1	3392
6	Ofla	18	125	3419	1	3562
7	Raya Azebo	29	105	2369	0	2505

This is not a geometrical data; therefore, it is not shown in map area

You can check the attributes of each data by **right click > Open Attribute Table**

6. Join your spread sheet data to the shape file of interest (In our case we joined "Zoonoses by 100000 pop" spread sheet to "study sites" shape file). To do this use the joins feature of qGIS.

   NB: Not every dataset you want to use comes as a shapefile, or in a spatial format. Often the data would come as a table or a spreadsheet and there could be a need to link it with your existing spatial data for use in your map. This operation is known as a **Table Join**. A unique

code is needed to be used to join to spatial data, “WOREDANAME” in our case.

7. To join a spreadsheet, double-click to open properties of the study sites layer (study sites) and select Joins from menu at the left-hand side.

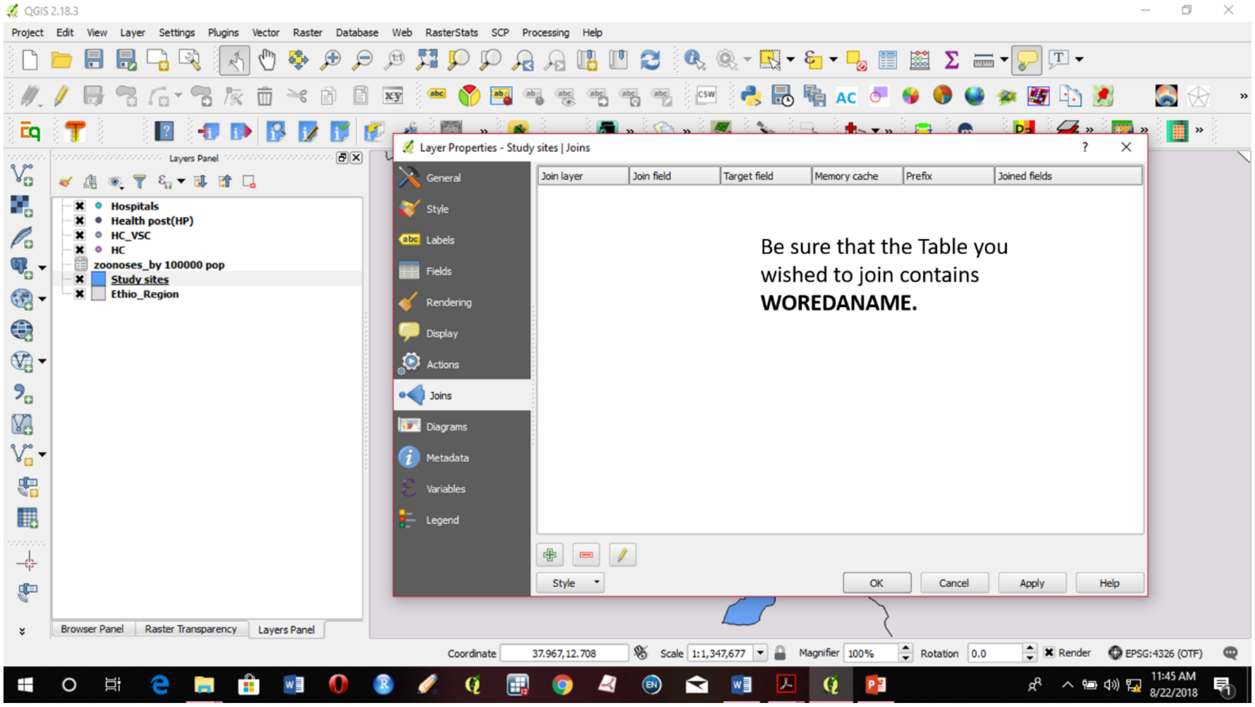

Click **plus (+)** sign to select a layer. Select the related field as follows and hit OK.

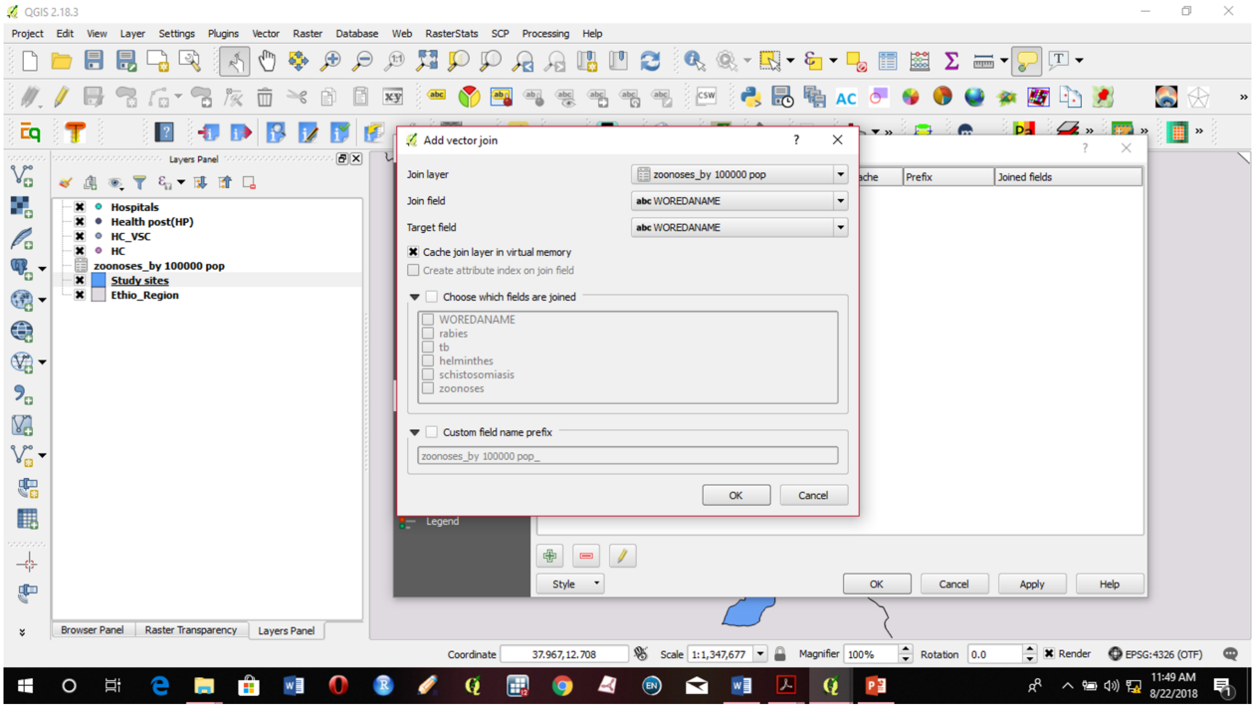

Now you have successfully joined your data set. To check the joining: Right click on Study sites > Open Attribute Table. You will see additional columns in your spatial data (in our case, rabies, tb, helminthiasis, schistosomiasis and total zoonoses).

8. To map the distribution of zoonotic diseases, Double-click to the study sites layer to open the properties. In the following example we see the distribution and incidence rate map for rabies. It can be done in a similar approach for the other diseases.

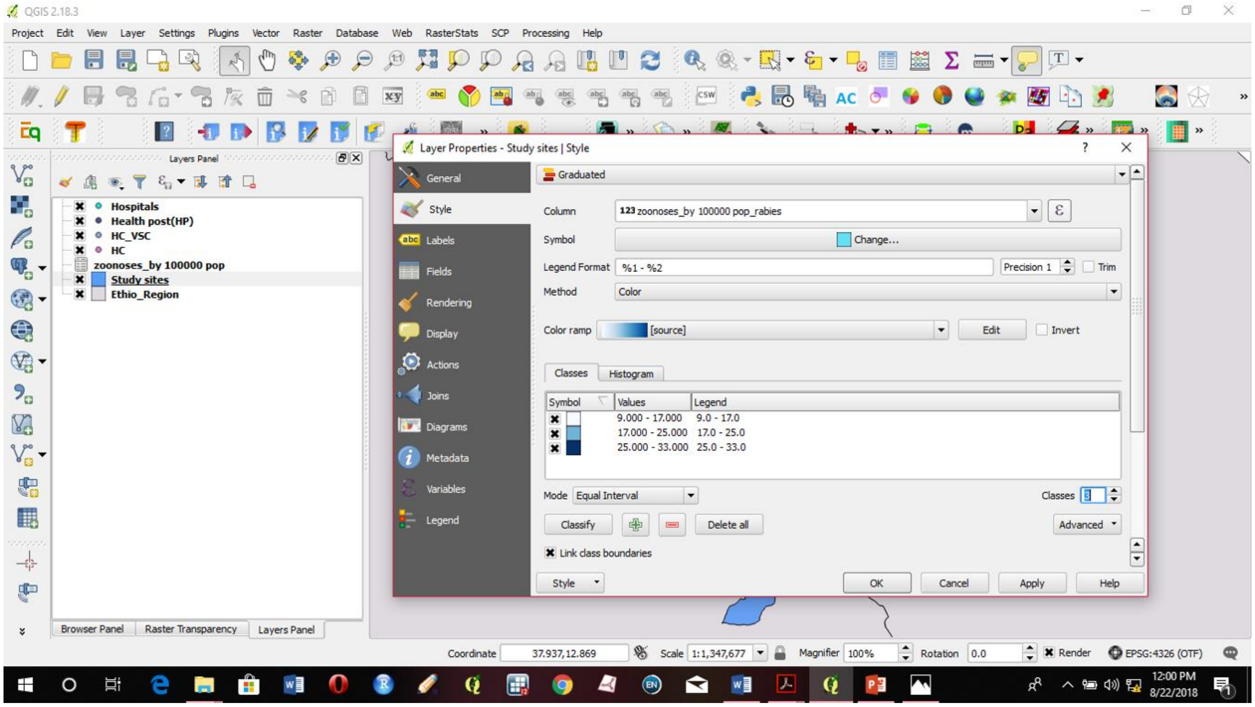

- Select Style from left-hand menu
- Select Graduated from the top dropdown menu
- Select zoonoses_by_100000 pop_rabies in column menu
- Select a color ramp you like from color ramp menu
- Select Quantile (Equal Count) from Mode and select classes (we choose 3) in classes menu
- Hit classify
- Change the values to how you think meaningful (we set the range 9.0 – 17.0; 17.1 – 25.0 and 25.1– 33.0)
- Hit okay to see your map!

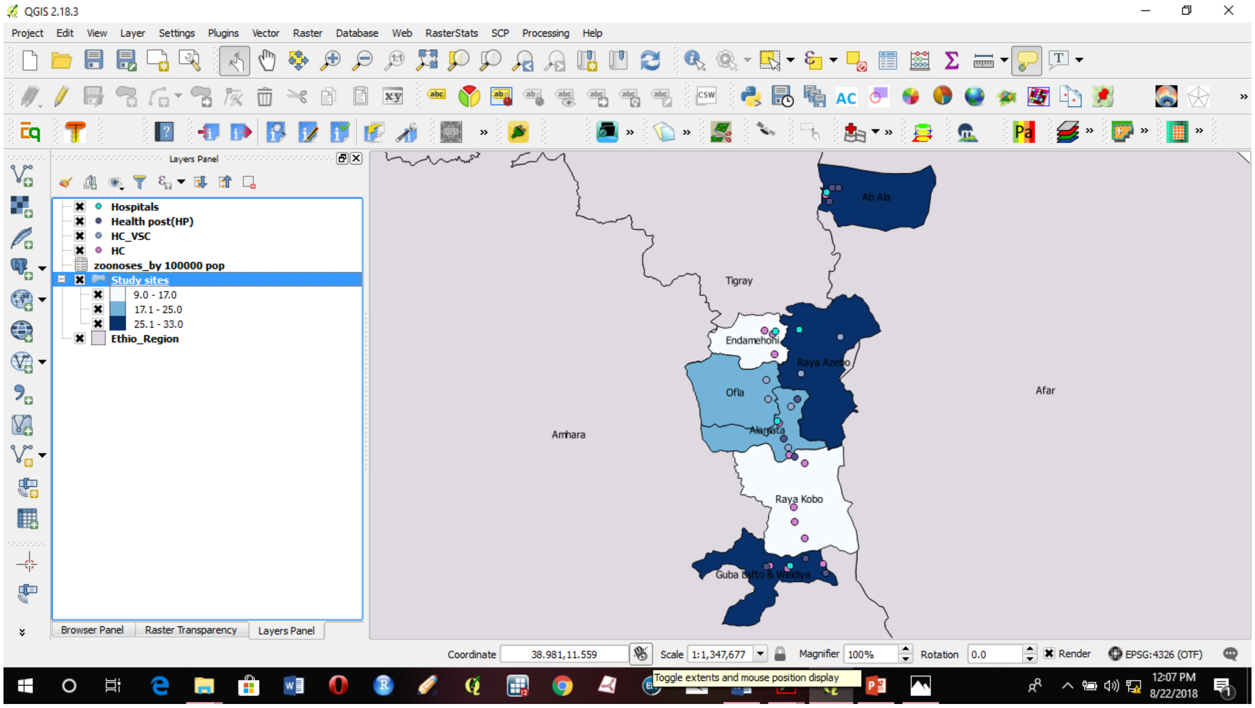

9. Export the distribution maps into image formats: click on project from the menu bar > New Print composer > When a dialog box appears, give a tile (e.g. Incidence rate of Rabies)

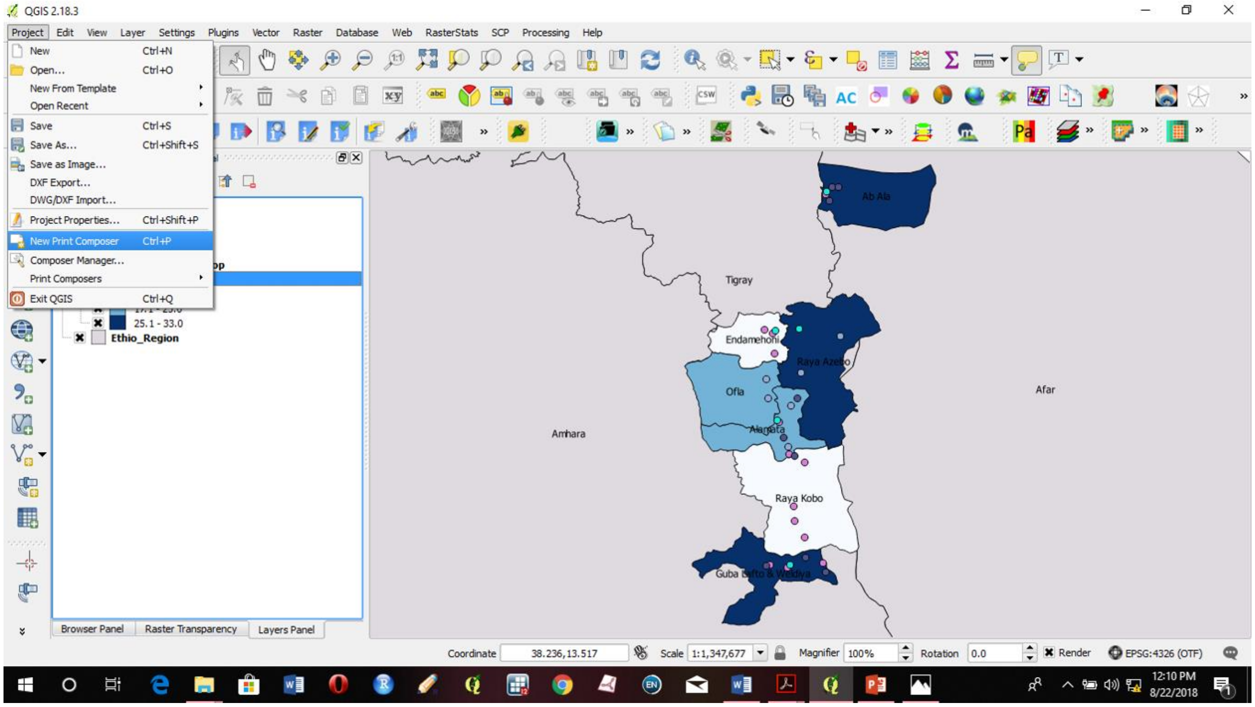

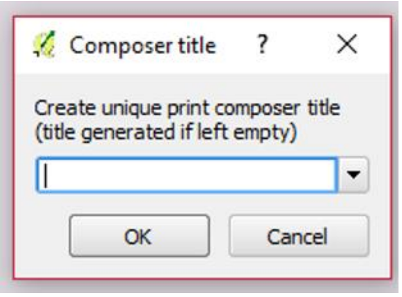

Following this a new Print composer window with the tile appears (in this case "Incidence rate of Rabies")

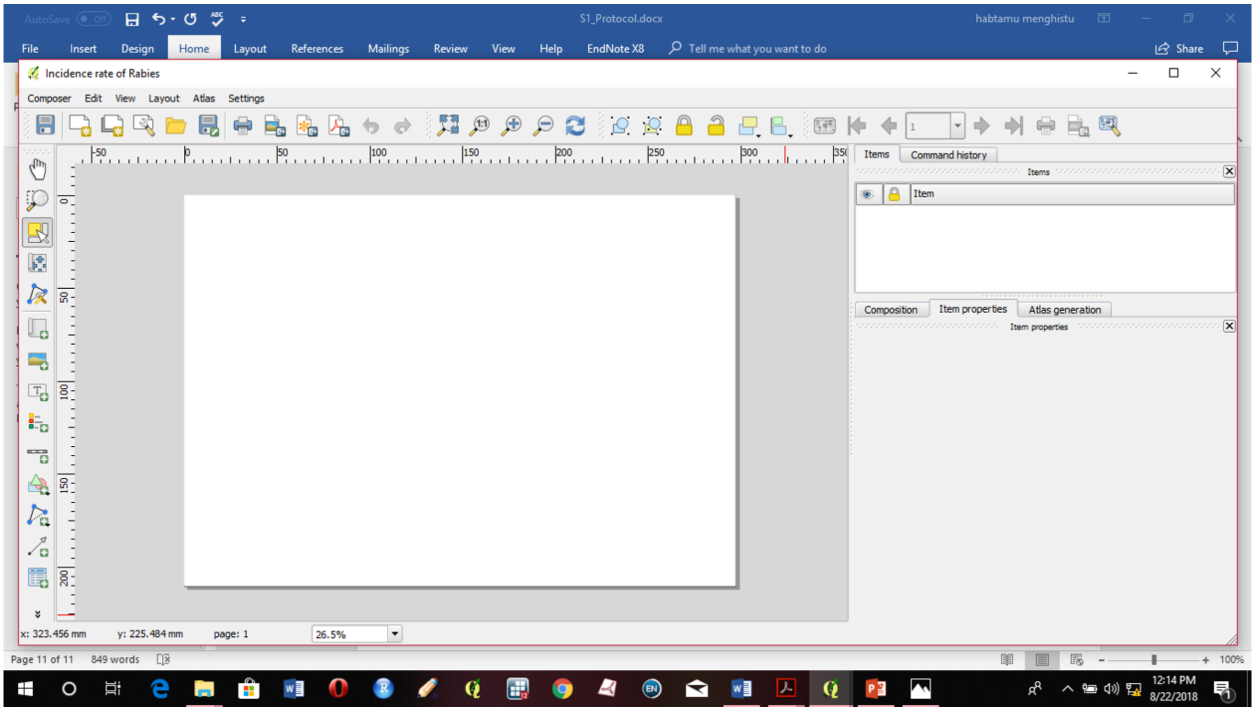

From the left menu click on "Add new Map" > Adjust the scale of the map using the Map Properties > Scale from the right side. Add legend (clicking on "Add New Legend"), Scalebar ("Add New Scalebar"), North Arrow ("Add Image") for decorating your map. You can edit the legend by going to legend items (unmark the "autoupdate" to do editing). Yu can also add title to your map.

Export your map by clicking on "Export as image" from the menu bar. Finally, your map will have the following look.

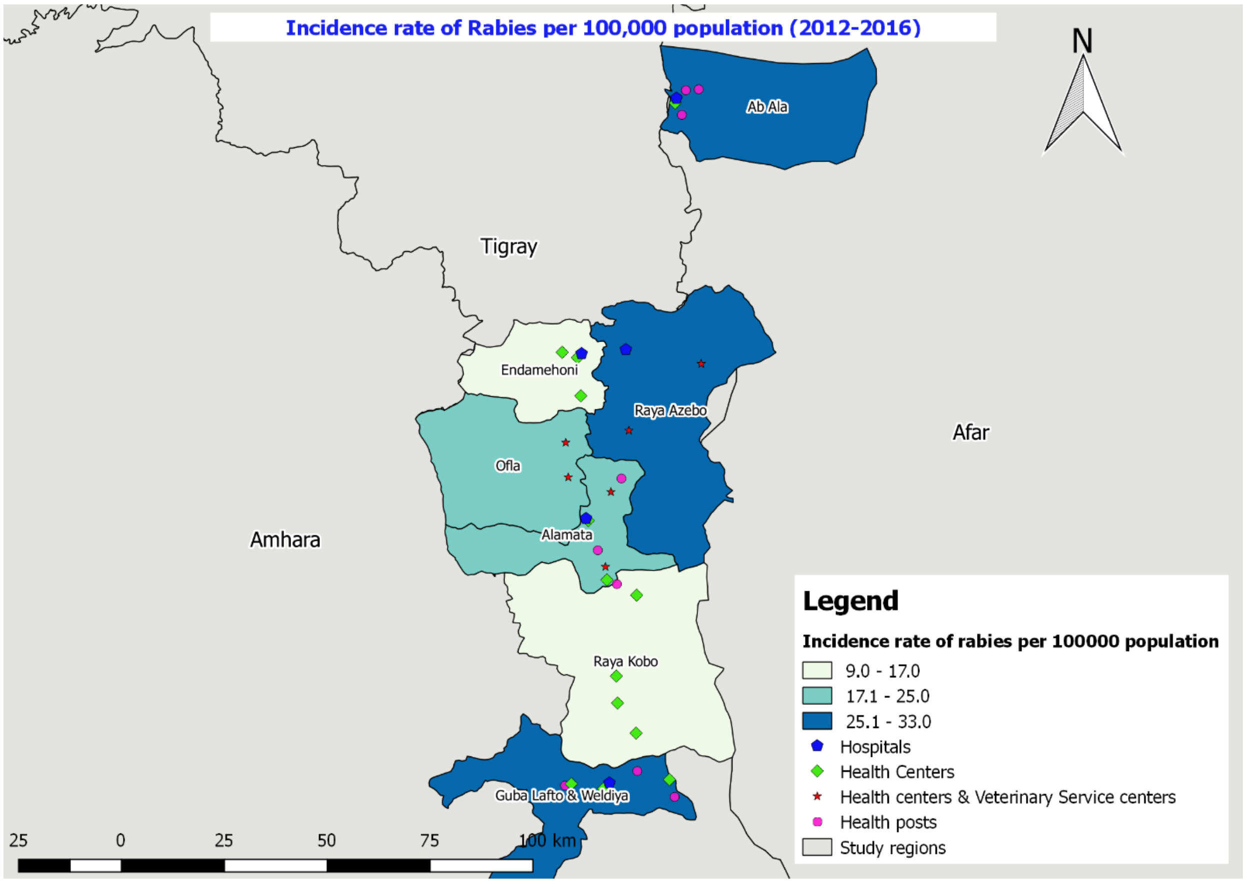

USEFUL LINKS:

Official QGIS User Guide:
https://docs.qgis.org/2.2/en/docs/index.html
Official QGIS Training Guide:
https://docs.qgis.org/2.2/en/docs/training_manual/
A Gentle Introduction to GIS:
https://docs.qgis.org/2.8/en/docs/gentle_gis_introduction/
Introduction to Mapping for Humanitarian Use:
http://www.refugeeinfoturkey.org/repo/Toolkit/CapacityBuilding/GIS/01-Introduction+to+GIS.pdf
